# Extended Variational Message Passing for Automated Approximate Bayesian Inference

**DOI:** 10.3390/e23070815

**Published:** 2021-06-26

**Authors:** Semih Akbayrak, Ivan Bocharov, Bert de Vries

**Affiliations:** 1 Department of Electrical Engineering, Eindhoven University of Technology, P.O. Box 513, 5600MB Eindhoven, The Netherlands; i.a.bocharov@tue.nl (I.B.); Bert.de.Vries@tue.nl (B.d.V.); 2 GN Hearing BV, JF Kennedylaan 2, 5612AB Eindhoven, The Netherlands

**Keywords:** Bayesian inference, variational inference, factor graphs, variational message passing, probabilistic programming

## Abstract

Variational Message Passing (VMP) provides an automatable and efficient algorithmic framework for approximating Bayesian inference in factorized probabilistic models that consist of conjugate exponential family distributions. The automation of Bayesian inference tasks is very important since many data processing problems can be formulated as inference tasks on a generative probabilistic model. However, accurate generative models may also contain deterministic and possibly nonlinear variable mappings and non-conjugate factor pairs that complicate the automatic execution of the VMP algorithm. In this paper, we show that executing VMP in complex models relies on the ability to compute the expectations of the statistics of hidden variables. We extend the applicability of VMP by approximating the required expectation quantities in appropriate cases by importance sampling and Laplace approximation. As a result, the proposed Extended VMP (EVMP) approach supports automated efficient inference for a very wide range of probabilistic model specifications. We implemented EVMP in the Julia language in the probabilistic programming package *ForneyLab.jl* and show by a number of examples that EVMP renders an almost universal inference engine for factorized probabilistic models.

## 1. Introduction

Probabilistic Programming Languages (PPL) and packages [1] have gained strong popularity over recent years since they support fast algorithm development through automating Bayesian inference in probabilistic models. Many of these PPLs [2,3,4,5] are based on numerical approximation methods, which leads to inexact inference results, even if the model comprises conjugate factor pairs and exact inference is achievable. Moreover, although a majority of popular PPLs scale well to processing large data sets due to their stochastic inference settings [6], they tend to execute very slowly for certain types of structured dynamic models, such as state space models. Alternatively, some PPLs that execute inference by message passing in a factor graph [7,8] provide efficient inference performance by exploiting factorization and conjugacy between exponential family-based distribution pairs in the model. In particular the Variational Message Passing (VMP) [9,10] algorithm has gained a good reputation, as it supports efficient inference for conjugate factor pairs in factorized probabilistic models. Unfortunately, non-conjugate factor pairs complicate the automated estimation of posterior distributions, due to intractability of the normalization constants. Likewise, non-linear deterministic relations between model variables often create non-conjugate pairings and thus obstruct the message-passing-based inference mechanism.

This paper proposes an Extended VMP (EVMP) algorithm to support automated efficient inference on a wide class of models that contain both non-conjugate relations between factor pairs and deterministic, possibly non-linear factor nodes. In our solution proposal, the regular VMP algorithm constructs the functional forms of the messages. These functional forms contain expectations of functions of hidden variables. In the case that these expectation quantities cannot be evaluated to a closed-form expression, we estimate them by Importance Sampling (IS) [11], which is a well-known Monte Carlo method that approximates intractable posteriors by a set of weighted samples and estimates expectations over this sample set. We also make use of Laplace approximation ([12], Section 4.4) with support by automatic differentiation tools (autodiff) [13] in appropriate cases to approximate posteriors by normal distributions, which allows us to calculate the expectations over the approximating normal distribution. Our proposal leads to an efficient, automatable message-passing framework that removes most model specification limitations.

In Section 2, we start with a review of factor graphs and the VMP algorithm. Next, we specify the proposed Extended VMP algorithm in Section 3. In order to keep the paper readable, both for the advanced researcher and someone who just needs the results, we defer detailed discussions and derivations of the key equations in EVMP to Appendix A and Appendix B. We implemented EVMP in the Julia package *ForneyLab.jl* [8,14]. In Section 4 we present several comparative experiments of EVMP in ForneyLab vs. *Turing.jl*, which is an alternative state-of-the-art Julia-based PPL that focuses on Monte Carlo methods for inference. We show that EVMP transforms ForneyLab into an almost universally applicable inference engine, while retaining computational efficiency, due to its library of closed-form message passing rules. An extensive comparison to related work is presented in Section 5.

## 2. Problem Statement

### Variational Message Passing on Forney-Style Factor Graphs

We assume a probabilistic model p(y,z) with a given set of observations $y=y_{1:N}=\left\{y_{1},\ldots ,y_{N}\right\}$ and a set of latent variables $z=z_{1:M}=\left\{z_{1},\ldots ,z_{M}\right\}$. Bayesian inference in this model relates to evaluating the following posterior:

$$p\left(z|y\right)=\frac{p(y,z)}{\int p(y,z)dz}\;,$$

which relies on evaluating the model evidence ∫p(y,z)dz. Unfortunately, due to the computational complexity of evaluating the integral for the evidence, exact Bayesian inference is achievable only for a limited set of probabilistic models. Alternatively, inference can be executed by minimization of a variational objective called free energy.

(1) $$\begin{array}{cc} F[q] & =E_{q}\left[\log\frac{q(z)}{p(y,z)}\right]\\ \; & =D_{KL}\left[q\left(z\right)||p\left(z|y\right)\right]-\log p\left(y\right), \end{array}$$

where $E_{q}\left[\cdot \right]$ stands for the expectation with respect to q(z) and $D_{KL}\left[\cdot ||\cdot \right]$ denotes a Kullback–Leibler divergence. (In this paper, we denote the expected value ∫q(x)f(x)dx of function *f* with respect to distribution *q* both by $E_{q}\left[f(x)\right]$ and ${\langle f(x)\rangle }_{q}$.) The KL divergence is greater or equal to zero for any distribution q(z) and $D_{KL}\left[q\left(z\right)||p\left(z|y\right)\right]=0$ if and only if q(z)=p(z|y). As a result, minimizing the free energy with respect to *q* leads both to an approximate posterior

$$q^{*}\left(z\right)=\arg\max_{q}F\left[q\right]\approx p\left(z|y\right)$$

and an upper bound $F[q^{*}]$ on the negative log-evidence. In practice, minimization of F is often greatly alleviated by assuming a mean-field constraint, i.e., a fully factorized posterior $q\left(z_{1:M}\right)=\prod_{i=1}^{M}q\left(z_{i}\right)$.

Variational inference on factorized models p(y,z) with the mean-field assumption for *q* leads to an automatable algorithm called Variational Message Passing (VMP) [9,10]. VMP can be visualized by representing the model as a graph and interpreting the VMP update equations as messages.

**Figure 1:**
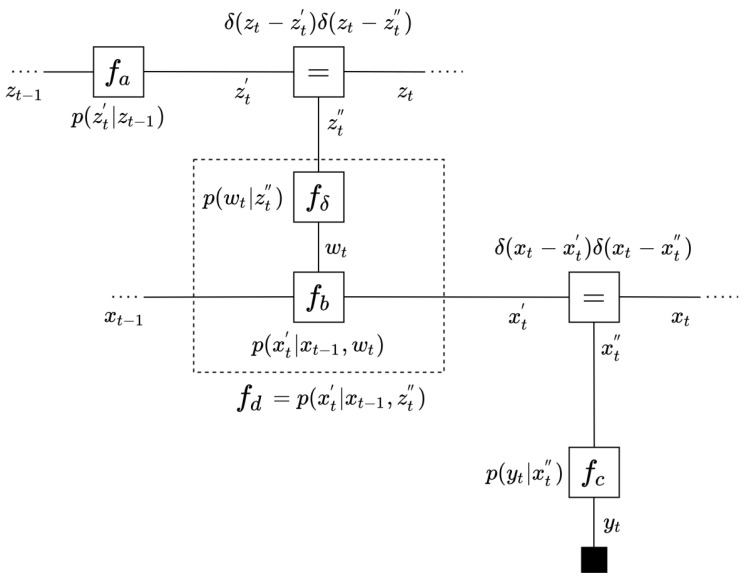
An FFG representation of one time step of a state space model. In FFGs, factors represent (conditional) distributions. Here, $f_{a}$, $f_{b}$ and $f_{c}$ are soft factors that each represent an exponential family distribution. On the other hand, $f_{\delta }=\delta \left(w_{t}-g\left(z_{t}\right)\right)$ represents a deterministic factor, where g(·) is a deterministic function. It is possible to compose factors and consider them as a single unit. In this example, $f_{d}$, visualized by a dashed box, stands for the composition of $f_{\delta }$ and $f_{b}$. It is a notational convention to visualize observed values ($y_{t}$) by a small black node.

In this paper, we favor a *Forney-style* Factor Graph (FFG) representation to visualize the factorization properties of probabilistic models and inference by message passing [15]. FFGs are undirected graph representations of factorized probabilistic models in which the conditional distributions are represented by nodes and the variables are associated with edges that connect the nodes. Besides visualizing the factorization properties of probabilistic models, FFGs also provide a formal framework for message passing-based inference in probabilistic models. In the FFGs that we discuss here, we distinguish three types of factors (nodes): soft factors, deterministic factors and equality factors. Throughout the paper, a soft factor represents an Exponential Family (EF) distribution (see (6) for definition) such as a Gaussian, Bernoulli, Gamma or Categorical distribution. Deterministic factors hold deterministic mappings of variables; in particular, we will use the relation $f_{\delta }\left(x,z\right)=p\left(x|z\right)=\delta \left(x-g\left(z\right)\right)$, where g(·) is a deterministic function. Lastly, equality factors are used to circumvent the constraint that an edge (representing a variable *z*) can only be connected to maximally two factors. In an FFG representation, this problem is resolved by adding variable copies $z^{\prime }$ and $z^{\prime \prime }$ and constraining the beliefs over these copy variables through an equality factor $f_{=}\left(z,z^{\prime },z^{\prime \prime }\right)=\delta \left(z-z^{\prime }\right)\delta \left(z-z^{\prime \prime }\right)$. As an example, the FFG for one time step for a hierarchical state-space model is visualized in Figure 1. In this graph, the factors $f_{a}\left(z_{t}^{\prime },z_{t-1}\right)=p\left(z_{t}^{\prime }|z_{t-1}\right)$, $f_{b}\left(x_{t}^{\prime },x_{t-1},w_{t}\right)=p\left(x_{t}^{\prime }|x_{t-1},w_{t}\right)$, $f_{c}\left(y_{t},x_{t}^{\prime \prime }\right)=p\left(y_{t}|x_{t}^{\prime \prime }\right)$ are encoded by EF distributions. The factor $f_{\delta }\left(w_{t},z_{t}^{\prime \prime }\right)=p\left(w_{t}|z_{t}^{\prime \prime }\right)=\delta \left(w_{t}-g\left(z_{t}^{\prime \prime }\right)\right)$, for a given function g(·), represents a non-linear deterministic relation. An interesting property of FFGs is the hierarchical composition: we can create new “higher level” nodes by enclosing a set of connected nodes in a box and integrating out the internal variables in the box. For instance, the *composite* node $f_{d}$ can be created through the following:

$$\begin{array}{cc} f_{d}\left(x_{t}^{\prime },x_{t-1},z_{t}^{\prime \prime }\right) & =p(x_{t}^{\prime }|x_{t-1},z_{t}^{\prime \prime })\\ \; & =\int p\left(x_{t}^{\prime }|x_{t-1},w_{t}\right)\delta \left(w_{t}-g\left(z_{t}^{\prime \prime }\right)\right)dw_{t}\\ \; & =\int f_{b}\left(x_{t}^{\prime },x_{t-1},w_{t}\right)f_{\delta }\left(w_{t},z_{t}^{\prime \prime }\right)dw_{t} \end{array}$$

For a more detailed introduction to FFGs, we refer to [15,16].

**Figure 2:**
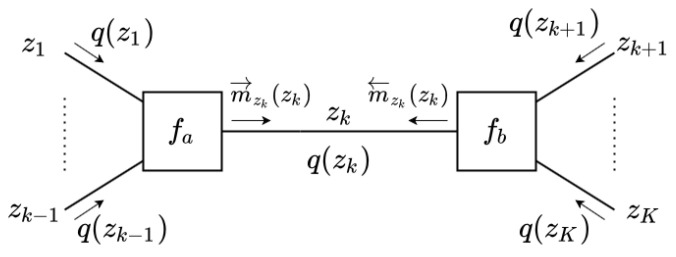
FFG representation for edge $z_{k}$ with connected nodes $f_{a}$ and $f_{b}$.

Aside from visualization, FFGs also serve to formalize message-passing-based inference in probabilistic models, and VMP on FFGs realizes coordinate-descent optimization of the free energy functional (1). Coordinate-descent optimization of the free energy refers to iterative updates of the variational factors one at a time while keeping the other factors fixed [12,17]. To illustrate, let us optimize F with respect to $q(z_{k})$ for the system $f_{a}\left(z_{1:k}\right)f_{b}\left(z_{k:K}\right)$ (see Figure 2). First, we decompose the free energy as follows:

$$\begin{array}{c} F=\tilde{F}+\underset{F_{k}}{\underbrace{E_{q(z_{1:K})}\left[\log\frac{q(z_{k})}{f_{a}\left(z_{1:k}\right)f_{b}\left(z_{k:K}\right)}\right]}}, \end{array}$$

where $\tilde{F}$ holds terms that are not a function of variable $z_{k}$. The term $F_{k}$ can be re-arranged as follows:

(2) $$\begin{array}{cc} F_{k} & =E_{q(z_{k})}\left[\log\frac{q(z_{k})}{\exp\left(E_{q(z_{1:k-1})}\left[\log f_{a}\left(z_{1:k}\right)\right]\right)\exp\left(E_{q(z_{k+1:K})}\left[\log f_{b}\left(z_{k:K}\right)\right]\right)}\right]\;, \end{array}$$

so it follows that $F_{k}$ is minimized when $q(z_{k})$ is set proportional to the denominator in (2) [12,17]. In addition, notice that the terms with the local factors $f_{a}$ and $f_{b}$ are uncoupled, which paves the way for a message-passing interpretation of coordinate-descent variational inference. As a result, provided that $f_{a}$ and $f_{b}$ are not deterministic factors, the VMP algorithm proceeds by repeating the following four steps until convergence [10]:

1. Choose a variable $z_{k}$ from the set $z_{1:K}$.
2. Compute the incoming messages.
  (3) $$\begin{array}{cc} {\vec{m}}_{z_{k}}\left(z_{k}\right) & \propto \exp\left({\langle \log f_{a}\left(z_{1:k}\right)\rangle }_{q(z_{1:k-1})}\right) \end{array}\;\begin{array}{cc} {\overset{\leftarrow }{m}}_{z_{k}}\left(z_{k}\right) & \propto \exp\left({\langle \log f_{b}\left(z_{k:K}\right)\rangle }_{q(z_{k+1:K})}\right) \end{array}$$
3. Update the posterior.
  (4) $$\begin{array}{c} q\left(z_{k}\right)=\frac{{\vec{m}}_{z_{k}}\left(z_{k}\right){\overset{\leftarrow }{m}}_{z_{k}}\left(z_{k}\right)}{\int {\vec{m}}_{z_{k}}\left(z_{k}\right){\overset{\leftarrow }{m}}_{z_{k}}\left(z_{k}\right)dz_{k}}. \end{array}$$
4. Update the local free energy (for performance tracking), i.e., update all terms in F that are affected by the update (4):
  (5) $$\begin{array}{c} F_{k}={\langle \log\frac{q(z_{k})}{f_{a}\left(z_{1:k}\right)f_{b}\left(z_{k:K}\right)}\rangle }_{q(z_{1:K})}. \end{array}$$

As we see in (3), messages flow on edges in both directions. It is common parlance to call one of the messages the forward message (denoted by ${\vec{m}}_{z_{k}}\left(z_{k}\right)$) and the other the backward message (${\overset{\leftarrow }{m}}_{z_{k}}\left(z_{k}\right)$).

In this paper, the central problem is *how to execute the VMP update Equation* (3) *through* (5) *for a wide range of specifications for the factors $f_{a}$ and $f_{b}$*. In the next section, we specify the proposed Extended VMP (EVMP) solution. A more detailed derivation of the key equations of EVMP is provided in Appendix B.

## 3. Specification of EVMP Algorithm

Variational Message Passing is a fast, efficient and deterministic approximate inference algorithm. However, the applicability of VMP heavily relies on connected factors being conjugate pairs (see Appendix A). In contrast, Monte Carlo methods (see [18] for message-passing interpretation) are applicable to a wider range of models with non-conjugate factor pairs. Unfortunately, in comparison to VMP, Monte Carlo methods are considerably slower since they rely on stochastic simulations. As we elaborate in Section 5, the recent efforts to combine the best of Monte Carlo methods and variational inference predominantly focus on noisy gradient estimation of the free energy through Monte Carlo sampling and do not take the full advantage of deterministic message passing steps in inference.

In this section, we specify the EVMP algorithm, which combines the efficiency of VMP with the flexibility of the Laplace approximation and the universality of Monte Carlo methods. In the proposed EVMP algorithm, VMP constructs the functional forms of the messages while importance sampling and Laplace approximations are used to estimate the required expectations of statistical quantities if they are not available in closed form. We first specify the range of probability distribution types for factors, messages and posteriors. These different types are used to identify the specific calculation rules for updating the messages and posteriors in (3) and (4). We refer the interested reader to Appendix B for detailed derivations.

### 3.1. Distribution Types

We consider the following representation types for probability distributions in factors p(z), where *z* holds a variable.

(1) The standard *Exponential Family* (EF) of distributions, i.e., the following:
  (6) $$p\left(z\right)=h\left(z\right)\exp\left(\phi {\left(z\right)}^{⊺}\eta -A_{\eta }\left(\eta \right)\right),$$
  where h(z) is the base measure, ϕ(z) is the sufficient statistics vector, η is the natural parameters vector and $A_{\eta }\left(\eta \right)$ is the log-partition function.
(2) Distributions that are of the following exponential form:
  (7) $$p\left(z\right)\propto \exp\left(\phi {\left(g\left(z\right)\right)}^{⊺}\eta \right),$$
  where g(z) is a deterministic function. The key characteristic here is that ϕ(g(z)) is not recognized as a sufficient statistics vector for any of the standard EF distributions. We call this distribution type a *Non-Standard Exponential Family* (NEF) distribution. As we show in Section 3.6, this distribution type arises only in backward message calculations.
(3) A *List of Weighted Samples* (LWS), i.e., the following:
  (8) $$p\left(z\right):=\left\{\left(w^{\left(1\right)},z^{\left(1\right)}\right),\ldots ,\left(w^{\left(N\right)},z^{\left(N\right)}\right)\right\}\;.$$
(4) Deterministic relations are represented by *delta distributions*, i.e., the following:
  (9) p(x|z)=δ(x−g(z)).
  Technically, the equality factor f(x,y,z)=δ(z−x)δ(z−y) also specifies a deterministic relation between variables.

### 3.2. Factor Types

Factor types f(z) are represented by EF and delta distributions.

In a VMP setting, as discussed in this and previous papers on VMP, conjugate soft factors from the exponential family enjoy some computational advantages. As an extension to VMP, the EVMP algorithm inherits the same computational advantages for conjugate factor pairs. In order to automate and generalize the inference to custom non-conjugate soft factors, we compose a generic soft factor by a delta distribution (to describe a non-linear deterministic function) and a standard EF distribution. This decomposition relieves us from manually deriving VMP messages for each different soft factor specification. For a given composite node (delta + standard EF), the EVMP algorithm uses the predefined VMP messages for the standard EF component to compute messages around the composite node. As we will see, this formulation yields an almost generic inference procedure.

### 3.3. Message Types

Forward messages carry either an EF or an LWS distribution. Backward messages carry either an EF or an NEF distribution. This is an arbitrary choice in the sense that we only make this assignment to indicate that in the EVMP algorithm, two colliding messages in posterior calculations are not both of the LWS type nor both of the NEF type.

### 3.4. Posterior Types

The posteriors q(z) are represented by either the EF or LWS representations.

To summarize the terminology so far, we defined four distribution types: Standard EF (EF), Non-Standard EF (NEF), List of Weighted Samples (LWS) and delta distributions. The end user of our algorithm can design a model by using EF and delta distributions. Under the hood, messages may carry EF, NEF or LWS distributions to render the inference. As the output, the end user is provided with either the EF or LWS posteriors. Next, we discuss how posteriors, messages and free energies are computed in the EVMP algorithm. The different types can be used to identify which computational recipe applies. As an aside, Julia’s support for multiple dispatch in functions [14] makes this a very elegant mechanism that requires almost no if–then rules.

### 3.5. Computation of Posteriors

Here, we discuss how EVMP updates the posteriors in (4). In an FFG, computation of the posterior q(z) is realized by a multiplication of colliding forward and backward messages, respectively ${\vec{m}}_{z}\left(z\right)$ and ${\overset{\leftarrow }{m}}_{z}\left(z\right)$, followed by normalization. We distinguish four types of updates.

(1) In the case that the colliding forward and backward messages both carry EF distributions with the same sufficient statistics ϕ(z), then computing the posterior simplifies to a summation of natural parameters:
  $$\begin{array}{cc} {\vec{m}}_{z}\left(z\right) & \propto \exp\left(\phi {\left(z\right)}^{⊺}\eta_{1}\right)\\ {\overset{\leftarrow }{m}}_{z}\left(z\right) & \propto \exp\left(\phi {\left(z\right)}^{⊺}\eta_{2}\right)\\ q(z) & \propto {\vec{m}}_{z}\left(z\right)\cdot {\overset{\leftarrow }{m}}_{z}\left(z\right)\propto \exp\left(\phi {\left(z\right)}^{⊺}\left(\eta_{1}+\eta_{2}\right)\right). \end{array}$$
  In this case, the posterior q(z) will also be represented by the EF distribution type. This case corresponds to classical VMP with conjugate factor pairs.
(2) The forward message again carries a standard EF distribution. The backward message carries either an NEF distribution or a non-conjugate EF distribution.
  (a) If the forward message is Gaussian, i.e., ${\vec{m}}_{z}\left(z\right)=N\left(z;\mu_{1},V_{1}\right)$, we use a Laplace approximation to compute the posterior:
    (10) $$\begin{array}{cc} \mu & =\arg\max_{z}\left(\log{\vec{m}}_{z}\left(z\right)+\log{\overset{\leftarrow }{m}}_{z}\left(z\right)\right),\\ V & ={\left(-\nabla \nabla_{z}\left(\log{\vec{m}}_{z}\left(z\right)+\log{\overset{\leftarrow }{m}}_{z}\left(z\right)\right){|}_{z=\mu }\right)}^{-1}\\ q(z) & \propto {\vec{m}}_{z}\left(z\right)\cdot {\overset{\leftarrow }{m}}_{z}\left(z\right)=N\left(z;\mu ,V\right) \end{array}$$
  (b) Otherwise (${\vec{m}}_{z}\left(z\right)$ is not a Gaussian), we use Importance Sampling (IS) to compute the posterior:
    (11) $$\begin{array}{cc} z^{\left(1\right)},\ldots ,z^{\left(N\right)} & ∼{\vec{m}}_{z}\left(z\right),\\ {\tilde{w}}^{\left(i\right)} & ={\overset{\leftarrow }{m}}_{z}\left(z^{\left(i\right)}\right)\;\mathrm{for}\;i=1,\ldots ,N\\ w^{\left(i\right)} & ={\tilde{w}}^{\left(i\right)}/\sum_{j=1}^{N}{\tilde{w}}^{\left(j\right)}\;\mathrm{for}\;i=1,\ldots ,N\\ q(z) & \propto {\vec{m}}_{z}\left(z\right)\cdot {\overset{\leftarrow }{m}}_{z}\left(z\right)=\left\{\left(w^{\left(1\right)},z^{\left(1\right)}\right),\ldots ,\left(w^{\left(N\right)},z^{\left(N\right)}\right)\right\}. \end{array}$$
(3) The forward message carries an LWS distribution, i.e., the following:
  $${\vec{m}}_{z}\left(z\right):=\left\{\left(w_{1}^{\left(1\right)},z_{1}^{\left(1\right)}\right),\ldots ,\left(w_{1}^{\left(N\right)},z_{1}^{\left(N\right)}\right)\right\}\;,$$
  and the backward message carries either an EF or NEF distribution. In that case, the posterior computation refers to updating the weights in ${\vec{m}}_{z}\left(z\right)$ (see Appendix E):
  (12) $$\begin{array}{cc} {\tilde{w}}^{\left(i\right)} & =w_{1}^{\left(i\right)}{\overset{\leftarrow }{m}}_{z}\left(z_{1}^{\left(i\right)}\right)\;\mathrm{for}\;i=1,\ldots ,N\\ w^{\left(i\right)} & ={\tilde{w}}^{\left(i\right)}/\sum_{j=1}^{N}{\tilde{w}}^{\left(j\right)}\;\mathrm{for}\;i=1,\ldots ,N\\ z^{\left(1\right)},\ldots ,z^{\left(N\right)} & =z_{1}^{\left(1\right)},\ldots ,z_{1}^{\left(N\right)}\\ q(z) & \propto {\vec{m}}_{z}\left(z\right)\cdot {\overset{\leftarrow }{m}}_{z}\left(z\right)=\left\{\left(w^{\left(1\right)},z^{\left(1\right)}\right),\ldots ,\left(w^{\left(N\right)},z^{\left(N\right)}\right)\right\}. \end{array}$$

### 3.6. Computation of Messages

Here, we discuss how EVMP compute the messages (3). We specify different message calculation rules depending of the type of the factor.

**Figure 3:**
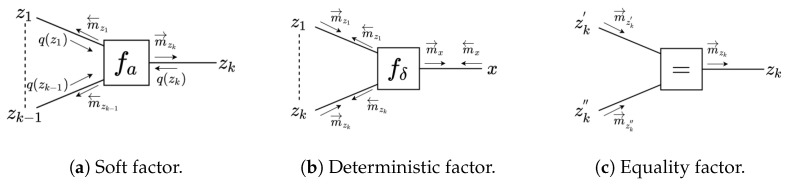
Different factor types for outgoing message computation rules.

(1) If factor $f_{a}\left(z_{1},z_{2},\ldots ,z_{k}\right)$ is a soft factor of the form (see Figure 3a)
  $$\begin{array}{c} f_{a}\left(z_{1:k}\right)=p\left(z_{k}|z_{1:k-1}\right)=h_{a}\left(z_{k}\right)\exp\left(\phi_{a}{\left(z_{k}\right)}^{⊺}\eta_{a}\left(z_{1:k-1}\right)-A_{a}\left(z_{1:k-1}\right)\right). \end{array}$$
  then the outgoing VMP message to $z_{k}$ is the following EF-distributed message:
  (13) $$\begin{array}{cc} {\vec{m}}_{z_{k}}\left(z_{k}\right) & \propto h_{a}\left(z_{k}\right)\exp\left(\phi_{a}{\left(z_{k}\right)}^{⊺}{\langle \eta_{a}\left(z_{1:k-1}\right)\rangle }_{q(z_{1:k-1})}\right). \end{array}$$
  If rather $z_{1}$ (or $z_{2},\ldots ,z_{k-1}$) than $z_{k}$ is the output variable of $f_{a}$, i.e., if the following is true:
  $$\begin{array}{c} f_{a}\left(z_{1:k}\right)=p\left(z_{1}|z_{2:k}\right)=h_{a}\left(z_{1}\right)\exp\left(\phi_{a}{\left(z_{1}\right)}^{⊺}\eta_{a}\left(z_{2:k}\right)-A_{a}\left(z_{2:k}\right)\right). \end{array}$$
  then the outgoing message to $z_{k}$ is either an EF or an NEF distribution of the following form:
  (14) $$\begin{array}{cc} {\overset{\leftarrow }{m}}_{z_{k}}\left(z_{k}\right) & \propto \exp\left({\langle \phi_{a}\left(z_{1}\right)\rangle }_{q(z_{1})}^{⊺}{\langle \eta_{a}\left(z_{2:k}\right)\rangle }_{q(z_{2:k-1})}-{\langle A_{a}\left(z_{2:k}\right)\rangle }_{q(z_{2:k-1})}\right). \end{array}$$
  In this last expression, we chose to assign a backward arrow to ${\overset{\leftarrow }{m}}_{z_{k}}\left(z_{k}\right)$ since it is customary to align the message direction with the direction of the factor, which in this case points to $z_{1}$.
  Note that the message calculation rule for ${\vec{m}}_{z_{k}}\left(z_{k}\right)$ requires the computation of expectation ${\langle \eta_{a}\left(z_{1:k-1}\right)\rangle }_{q(z_{1:k-1})}$, and for ${\overset{\leftarrow }{m}}_{z_{k}}\left(z_{k}\right)$ we need to compute expectations ${\langle \phi_{a}\left(z_{1}\right)\rangle }_{q(z_{1})}$ and ${\langle \eta_{a}\left(z_{2:k}\right)\rangle }_{q(z_{2:k-1})}$. In the update rules to be shown below, we will see these expectations of statistics of *z* appear over and again. In Section 3.8 we detail how we calculate these expectations and in Appendix A, we further discuss the origins of these expectations.
(2) In the case that $f_{\delta }$ is a deterministic factor (see Figure 3b):
  (15) $$\begin{array}{c} f_{\delta }\left(x,z_{1:k}\right)=p\left(x|z_{1:k}\right)=\delta \left(x-g\left(z_{1:k}\right)\right). \end{array}$$
  then the forward message from $f_{\delta }$ to *x* is of LWS type and is calculated as follows:
  (16) $$\begin{array}{cc} \; & {\vec{m}}_{x}\left(x\right)=\left\{\left(\frac{1}{N},g\left(z_{1:k}^{\left(1\right)}\right)\right),\ldots ,\left(\frac{1}{N},g\left(z_{1:k}^{\left(N\right)}\right)\right)\right\},\\ & \mathrm{where}\;z_{j}^{\left(i\right)}∼{\vec{m}}_{z_{j}}\left(z_{j}\right)\;\mathrm{for}\;j=1:k. \end{array}$$
  For the computation of the backward message toward $z_{k}$, we distinguish two cases:
  (a) If all forward incoming messages from the variables $z_{1:k}$ are Gaussian, we first use a Laplace approximation to obtain a Gaussian joint posterior $q\left(z_{1:k}\right)=N\left(z_{1:k};\mu_{1:k},V_{1:k}\right)$; see Appendix B.1.2 and Appendix B.2.2 for details. Then, we evaluate the posteriors for individual random variables, e.g., $q\left(z_{k}\right)=\int q\left(z_{1:k}\right)dz_{1:k-1}=N\left(z_{k};\mu_{k},V_{k}\right)$. Finally, we send the following Gaussian backward message:
    (17) $$\begin{array}{c} {\overset{\leftarrow }{m}}_{z_{k}}\left(z_{k}\right)\propto q\left(z_{k}\right)/{\vec{m}}_{z_{k}}\left(z_{k}\right). \end{array}$$
  (b) Otherwise (the incoming messages from the variables $z_{1:k}$ are not all Gaussian), we use Monte Carlo and send a message to $z_{k}$ as a NEF distribution:
    (18) $$\begin{array}{c} {\overset{\leftarrow }{m}}_{z_{k}}\left(z_{k}\right)\approx \frac{1}{N}\sum_{i=1}^{N}{\overset{\leftarrow }{m}}_{x}\left(g\left(z_{1:k-1}^{\left(i\right)},z_{k}\right)\right),\;\mathrm{where}\;z_{j}^{\left(i\right)}∼{\vec{m}}_{z_{j}}\left(z_{j}\right). \end{array}$$
    Note that if $f_{\delta }$ is a single input deterministic node, i.e., $f_{\delta }\left(x,z_{k}\right)=p\left(x|z_{k}\right)=\delta \left(x-g\left(z_{k}\right)\right)$, then the backward message simplifies to ${\overset{\leftarrow }{m}}_{z_{k}}\left(z_{k}\right)={\overset{\leftarrow }{m}}_{x}\left(g\left(z_{k}\right)\right)$ (Appendix B.1.1).
(3) The third factor type that leads to a special message computation rule is the equality node; see Figure 3c. The outgoing message from an equality node
  $$f_{=}\left(z,z^{\prime },z^{\prime \prime }\right)=\delta \left(z-z^{\prime }\right)\delta \left(z-z^{\prime \prime }\right)$$
  is computed by following the sum–product rule:
  (19) $$\begin{array}{cc} {\vec{m}}_{z_{k}}\left(z_{k}\right) & =\int \underset{\mathrm{node}\;\mathrm{function}}{\underbrace{\delta \left(z_{k}-z_{k}^{\prime }\right)\delta \left(z_{k}-z_{k}^{\prime \prime }\right)}}\underset{\mathrm{incoming}\;\mathrm{messages}}{\underbrace{{\vec{m}}_{z_{k}^{\prime }}\left(z_{k}^{\prime }\right){\vec{m}}_{z_{k}^{\prime \prime }}\left(z_{k}^{\prime \prime }\right)}}dz_{k}^{\prime }dz_{k}^{\prime \prime }\\ \; & ={\vec{m}}_{z_{k}^{\prime }}\left(z_{k}\right){\vec{m}}_{z_{k}^{\prime \prime }}\left(z_{k}\right). \end{array}$$

### 3.7. Computation of Free Energy

Here, we discuss how EVMP computes the FE update from (5). Note that the FE can be decomposed into a subtraction of energy and entropy terms:

(20) $$\begin{array}{cc} F_{k} & ={\langle \log\frac{q(z_{k})}{f_{a}\left(z_{1:k}\right)f_{b}\left(z_{k:K}\right)}\rangle }_{q(z_{1:K})}\\ \; & =\underset{\left(\mathrm{average}\right)\;\mathrm{energy}\;U_{a}+U_{b}}{\underbrace{{\langle \log\frac{1}{f_{a}\left(z_{1:k}\right)f_{b}\left(z_{k:K}\right)}\rangle }_{q(z_{1:K})}}}-\underset{\mathrm{entropy}\;H_{k}}{\underbrace{{\langle \log\frac{1}{q(z_{k})}\rangle }_{q(z_{k})}}} \end{array}$$

These energy and entropy terms can be evaluated because $f_{a}\left(z_{1:k}\right)f_{b}\left(z_{k:K}\right)$ contains only factors that are defined in the generative model and $q(z_{1:K})$ is also accessible as a result of variational inference. Thus, we evaluate the FE by evaluating the energy and entropy terms separately.

For an EF-encoded soft factor

$$f_{a}\left(z_{1:k}\right)=p\left(z_{k}|z_{1:k-1}\right)=h_{a}\left(z_{k}\right)\exp\left(\phi_{a}{\left(z_{k}\right)}^{⊺}\eta_{a}\left(z_{1:k-1}\right)-A_{\eta }\left(\eta_{a}\left(z_{1:k-1}\right)\right)\right)\;,$$

the energy over the factor $f_{a}$ evaluates to

$$\begin{array}{cc} U_{a} & =-{\langle \log(f_{a}\left(z_{1:k}\right))\rangle }_{q(z_{1:k})}\\ \; & =-{\langle \log(h_{a}\left(z_{k}\right))\rangle }_{q(z_{k})}-{\langle \phi_{a}\left(z_{k}\right)\rangle }_{q(z_{k})}^{⊺}{\langle \eta_{a}\left(z_{1:k-1}\right)\rangle }_{q(z_{1:k-1})}+{\langle A_{\eta }\left(\eta_{a}\left(z_{1:k-1}\right)\right)\rangle }_{q(z_{1:k-1})}. \end{array}$$

The entropy terms only need to be evaluated for variables *z* that are not associated with output edges of deterministic nodes. In that case, we calculate the entropy of q(z) as follows:

1. If q(z) is a represented by a standard EF distribution, i.e.,
  $$q\left(z\right)=h\left(z\right)\exp\left(\phi {\left(z\right)}^{⊺}\eta -A_{\eta }\left(\eta \right)\right),$$
  then
  $$\begin{array}{c} H_{z}=-{\langle \log(h(z))\rangle }_{q(z)}-{\langle \phi (z)\rangle }_{q(z)}^{⊺}\eta +A_{\eta }\left(\eta \right). \end{array}$$
2. Otherwise, if q(z) is represented by a LWS, i.e.,
  $$q\left(z\right):=\left\{\left(w^{\left(1\right)},z^{\left(1\right)}\right),\ldots ,\left(w^{\left(N\right)},z^{\left(N\right)}\right)\right\},$$
  then
  $$\begin{array}{c} H_{z}={\hat{H}}_{z}^{1}+{\hat{H}}_{z}^{2}, \end{array}$$
  where ${\hat{H}}_{z}^{1}$ and ${\hat{H}}_{z}^{2}$ are evaluated as discussed in (A43) and (A45).

### 3.8. Expectations of Statistics

In many of the above computations for messages, posteriors and free energies, we need to compute certain expectations of statistics of *z*, e.g., the computation of the forward message in (13) requires evaluation of ${\langle \eta_{a}\left(z_{1:k-1}\right)\rangle }_{q(z_{1:k-1})}$. Here, we discuss how EVMP evaluates these expectations. Let us denote a statistic of random variable *z* by Φ(z) and assume we are interested in the expected value ${\langle \Phi (z)\rangle }_{q(z)}$. The calculation rule depends on the type of q(z):

(1) We have two cases when q(z) is coded as an EF distribution, i.e.,
  $$q\left(z\right)=h\left(z\right)\exp\left(\phi {\left(z\right)}^{⊺}\eta -A_{\eta }\left(\eta \right)\right):$$
  (a) If Φ(z)∈ϕ(z), i.e., the statistic Φ(z) matches with elements of the sufficient statistics vector ϕ(z), then ${\langle \Phi (z)\rangle }_{q(z)}$ is available in closed form as the gradient of the log-partition function (this is worked out in Appendix A.1.1, see (A14) and (A15)):
    $${\langle \Phi (z)\rangle }_{q(z)}\in \nabla_{\eta }A_{\eta }\left(\eta \right).$$
  (b) Otherwise (Φ(z)∉ϕ(z)), then we evaluate
    $${\langle \Phi (z)\rangle }_{q(z)}\approx \frac{1}{N}\sum_{i=1}^{N}\Phi \left(z^{\left(i\right)}\right)\;,$$
    where $z^{\left(i\right)}∼q\left(z\right)$.
(2) In case q(z) is represented by a LWS, i.e., the following:
  $$q\left(z\right)=\left\{\left(w^{\left(1\right)},z^{\left(1\right)}\right),\ldots ,\left(w^{\left(N\right)},z^{\left(N\right)}\right)\right\}\;,$$
  then, we evaluate the following:
  $${\langle \Phi (z)\rangle }_{q(z)}\approx \sum_{i=1}^{N}w^{\left(i\right)}\Phi \left(z^{\left(i\right)}\right)\;.$$

### 3.9. Pseudo-Code for the EVMP Algorithm

Section 3.1, Section 3.2, Section 3.3, Section 3.4, Section 3.5, Section 3.6, Section 3.7 and Section 3.8 provide a recipe for almost universal evaluation of variational inference in factor graphs. We use classical VMP with closed-form solutions when possible, and resort to Laplace or IS approximations when needed. We now summarize the EVMP algorithm by a pseudo-code fragment in Algorithm 1. We use the following notation: $V=V_{f}\cup V_{\delta }\cup V_{=}$ is the set of factor nodes (vertices), where $V_{f}$, $V_{\delta }$, $V_{=}$ stand for the subsets of soft factor nodes, deterministic nodes and equality nodes, respectively. *E* is the set of edges that connect the nodes. G=(V,E) represents the entire factor graph. $h_{1:M+L}=z_{1:M}\cup x_{1:L}$ is the set of hidden variables, where $x_{1:L}$ are the variables at the output edges of deterministic nodes. $z_{1:M}$ are also associated with edges in *E*, but in contrast to $x_{1:L}$, $z_{1:M}$ are not output edges of deterministic nodes.

For structured factorizations, the overall structure remains the same, but messages and posteriors are calculated for sub-graphs instead of single random variables.

An example to illustrate the calculation of messages and posteriors in the EVMP algorithm is provided in Appendix F.
**Algorithm 1** Extended VMP (Mean-field assumption)**Require:** G=(V,E), $h_{1:M+L}$, $N_{iterations}$, $q_{initial}$ **for** j=1⋯M+L **do**  initialize posteriors $q(h_{j})$ using $q_{initial}$ **end for** **for** i = 1...$N_{iterations}$ **do**  Set Free Energy F=0  **for** j=1⋯M+L **do**   Calculate messages ${\vec{m}}_{h_{j}}\left(h_{j}\right)$ and ${\overset{\leftarrow }{m}}_{h_{j}}\left(h_{j}\right)$ using Section 3.6   Calculate posterior $q(h_{j})$ using Section 3.5   **if** $h_{j}\in z_{1:M}$ **then**    Calculate entropy $H_{h_{j}}$ using Section 3.7    Update Free Energy $F=F-H_{h_{j}}$   **end if**  **end for**  **for all** $v\in V_{f}$ **do**   Calculate energy $U_{v}$ using Section 3.7   Update Free Energy $F=F+U_{v}$  **end for**  **return** q(h) **for all** $h\in h_{1:M+L}$ and F **end for**

## 4. Experiments

We illustrate EVMP-based inference on three different applications (code for experiments can be found at https://github.com/biaslab/ExtendedVMP (accessed on 25 June 2021)). For each application, we show the favorable features of EVMP together with its shortcomings in comparison to Turing [5], which is a general purpose Julia probabilistic programming package.

### 4.1. Filtering with the Hierarchical Gaussian Filter

The Hierarchical Gaussian Filter (HGF) [19,20] is a popular generative model in the neuroscience community. The HGF consists of a Gaussian random walk model, where the variance of the Gaussian is a nonlinear function of the state of the next higher layer, that in turn evolves according to a Gaussian random walk, an so on. Due to the nonlinear link between the layers, classical VMP rules do not have a closed-form solution. While in principle, variational updates through Laplace approximation can be manually derived for the HGF model [19], automatically generated EVMP update rules alleviate the need for cumbersome and error-prone manual derivations.

The 2-layer HGF model is defined as

(21a) $$\begin{array}{cc} z_{t} & ∼N(z_{t-1},\sigma_{z}^{2}) \end{array}$$

(21b) $$\begin{array}{cc} w_{t} & =\exp\left(z_{t}\right) \end{array}$$

(21c) $$\begin{array}{cc} x_{t} & ∼N(x_{t-1},w_{t}) \end{array}$$

(21d) $$\begin{array}{cc} y_{t} & ∼N(x_{t},\sigma_{y}^{2})\;. \end{array}$$

**Figure 4:**
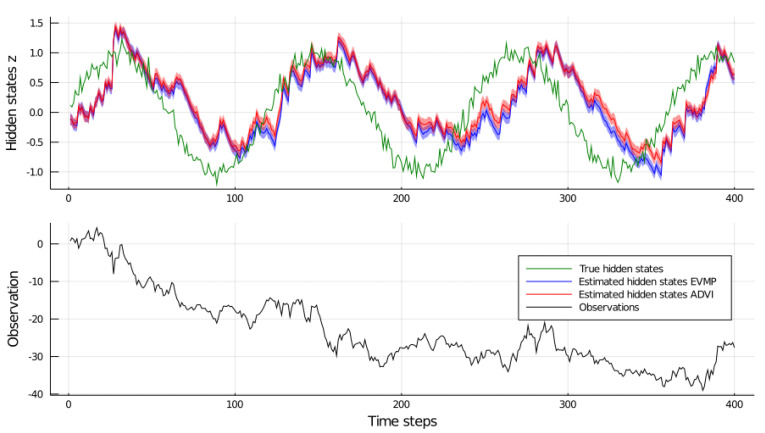
Above: Hidden states $z_{1:400}$ and their estimates (ribbon is one variance). The estimates of ForneyLab’s Extended VMP are designated by blue while the estimates of Turing’s ADVI are marked by red. Below: Observed synthetic data.

For this experiment, we generated T=400 data points by the following process. First, we generated noisy hidden states using $z_{t}∼N\left(\sin\left(\frac{\pi }{60}t\right),0.01\right),t=1\cdots 400$. Next, we generated observations following model (21a–d) with $\sigma_{y}^{2}=0.1$. The generated data set is visualized in (the lower subgraph of) Figure 4.

Next, we filtered the data set by a second HGF, also given by (21a–d) with priors $z_{0}∼N\left(0,1\right)$, $x_{0}∼N\left(0,1\right)$ and parameters $\sigma_{z}^{2}=\sigma_{y}^{2}=0.1$. We used EVMP to track the hidden states $z_{t}$ and $x_{t}$. All inference steps including the message passing schedule for filtering in the HGF are detailed in [19]. For each time step, EVMP was run for 10 iterations at each filtering step.

For comparison, we implemented a similar filtering procedure by Automatic Differentiation Variational Inference (ADVI) [21], executed by Julia’s *Turing.jl* [5] package. At each time step *t*, the priors over $z_{t-1}$ and $x_{t-1}$ are set to Gaussian distributions, the mean and variance parameters of which are determined by sampling from the variational posteriors at t−1. The only difference between the ForneyLab and Turing implementations, in terms of posterior distribution factorization, is that in Turing’s ADVI, we posit a fully factorized posterior distribution. This assumption decreases the number of parameters to be estimated via automatic differentiation and speeds up the inference procedure. On the other hand, pre-defined message passing rules in ForneyLab enable us to retain the dependency structure between $x_{t-1}$ and $x_{t}$ at time step *t* in exchange for almost no run-time loss. To be more precise, at time step *t*, we run inference on the following model: $q_{f}\left(z_{t-1}\right)p\left(z_{t}|z_{t-1}\right)\delta \left(w_{t}-\exp\left(z_{t}\right)\right)q_{f}\left(x_{t-1}\right)p\left(x_{t}|x_{t-1},w_{t}\right)p\left(y_{t}|x_{t}\right)$ where $q_{f}\left(z_{t-1}\right)$ and $q_{f}\left(x_{t-1}\right)$ are the posterior approximations from the previous time step. In ForneyLab, we run the inference with variational distribution $q\left(z_{t-1}\right)q_{f}\left(z_{t}\right)q\left(x_{t-1},x_{t}\right)$ with $q_{f}\left(x_{t}\right)=\int q\left(x_{t-1},x_{t}\right)dx_{t-1}$. We plot estimations for $q_{f}\left(z_{t}\right)$ in Figure 4. In ADVI, the variational distribution is $q\left(z_{t-1}\right)q_{f}\left(z_{t}\right)q\left(x_{t-1}\right)q_{f}\left(x_{t}\right)$. Once inference has completed, Turing allows drawing samples from the variational distribution. We then calculate the mean and variance of these samples to fit Gaussian distributions on $q_{f}\left(z_{t}\right)$ and $q_{f}\left(x_{t}\right)$.

The estimated tracks of $z_{t}$ are visualized in Figure 4. For both EVMP and ADVI, the estimated hidden states largely coincide. However, we observe that both methods capture the periodic character of the true hidden states $z_{1:400}$ with a delay. We believe that there are two plausible explanations for the delayed estimations: (1) in the model specification, we assume that the data generative process is not known fully. The variables $z_{1:400}$ are originally generated from a sinusoidal function of discrete time steps. However, in the model specification, we do not use this information; (2) in the model specification, we define a random walk over hidden variables $z_{1:400}$ that posits the mean of $z_{t}$ as $z_{t-1}$. Elaborating the latter factor, the random walk avoids a hidden variable $z_{t}$ to change drastically, compared to $z_{t-1}$ while $x_{t}$ forces $z_{t}$ to explain the volatility in the process. Reconciling the beliefs from $x_{t}$ and $z_{t-1}$, both Extended VMP and ADVI estimate $z_{t}$ with a delay.

In Turing’s ADVI procedure, we used 10 samples per iteration for gradient estimation and set the maximum number of iterations to 4000 per time step to be able to capture this periodic behavior. The overall inference is completed in roughly 1.5 min (this and furtherexperiments were carried out on a machine with the following specs: Julia 1.5.0, Turing v0.15.9, AMD Ryzen 7 3700X 3.6 GHz 8-Core CPU, 16 GB DDR4-3200 MHz RAM.). ForneyLab’s EVMP procedure, on the other hand, is able to perform inference in under 7 s on this time series; see Table 1. The speed of ForneyLab stems from the hybrid inference nature of EVMP. EVMP resorts to gradient-based optimization only to infer $q_{f}\left(z_{t}\right)$ and the sampling procedure is required only to estimate statistics related to $w_{t}$ to be used in the update steps of $q(x_{t-1},x_{t})$. In contrast, ADVI requires sampling and employs noisy gradients in the estimation of all the components of the variational distribution. This experiment validates EVMP as a fast automated variational inference solution for filtering in hierarchical dynamic models.

### 4.2. Parameter Estimation for a Linear Dynamical System

In this experiment, we focused attention on a system identification task in a Linear Dynamical System (LDS) [22,23]. An LDS is generally defined as

(22a) $$\begin{array}{cc} x_{t}|x_{t-1} & ∼N(Ax_{t-1},Q) \end{array}$$

(22b) $$\begin{array}{cc} y_{t}|x_{t} & ∼N(Bx_{t},R) \end{array}$$

where $y_{t}$ are observations and $x_{t}$ are hidden states.

In this experiment, we are interested in inferring the transition matrix *A* together with the hidden states from a set of observations. Manually derived closed-form solutions for the system identification task are available both in maximum likelihood estimation [24] and a variational Bayesian approximation [25] contexts. Nevertheless, the goal in this and other papers on probabilistic programming packages is to automatically infer posteriors over the hidden states and parameters without resorting to manual derivations. In principle, EVMP supports to infer the hidden states, *A*, *B*, *Q* and *R* concurrently. Of course, depending on specific circumstances such as system identifiability and the richness of the observed data, the performance may vary.

In order to execute our experiment, we first extend (22a,b) with a prior on *A* as follows:

(23a) $$\begin{array}{cc} a & ∼N(\mu_{a},V_{a}) \end{array}$$

(23b) $$\begin{array}{cc} A & =\mathrm{reshape}(a,(m,m)) \end{array}$$

(23c) $$\begin{array}{cc} x_{t}|x_{t-1} & ∼N(Ax_{t-1},Q) \end{array}$$

(23d) $$\begin{array}{cc} y_{t}|x_{t} & ∼N(Bx_{t},R) \end{array}$$

In (23a–d), *a* holds the vectorized representation of the transition matrix *A*. Note that (23b) can be written as follows:

p(A|a)=δ(A−reshape(a,(m,m))),

and through this manipulation we identify reshape(a,(m,m)) as the deterministic factor in (15). As a result, ForneyLab’s EVMP works out-of-the-box for inference of the transition matrix in (23a–d).

We first generated a data set of T=40 number of samples by running model (22a,b) with parameters $Q=0.01\times I_{2\times 2}$, $R=0.1\times I_{2\times 2}$, $B=I_{2\times 2}$ and $A=\left[\begin{array}{cc} 1.0 & 0.2\\ -0.5 & 0.8 \end{array}\right]$.

Next, we presented the data set to a second LDS model and aimed to infer posteriors over hidden states and transition matrix *A*. The prior on *a* was set to $a∼N(0_{4},I_{4\times 4})$ and all other parameters were set to the same values as in the data generation process.

**Figure 5:**
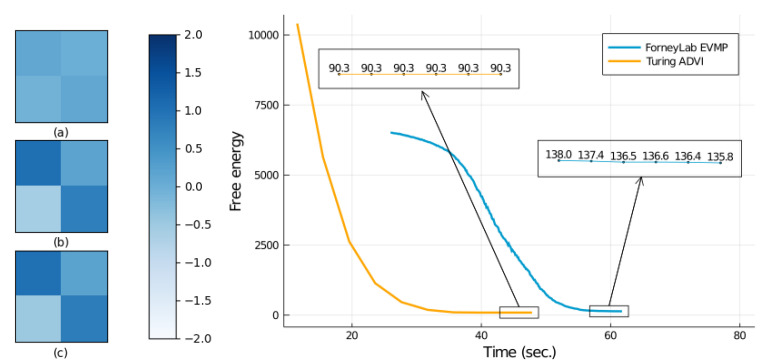
Free energy tracks for EVMP on the LDS transition matrix identification task. Left: (**a**) Mean estimate EVMP for the transition matrix *A* after 50 iterations, (**b**) mean estimate after 300 iterations, (**c**) true transition matrix *A* that was used to generate the synthetic data. Right: Free energy tracks by ForneyLab’s EVMP and Turing’s ADVI procedures.

We compared the performance of ForneyLab’s EVMP with Turing’s ADVI and NUTS (No U-Turn Sampler, a Hamiltonian Monte Carlo sampling-based inference method) [26] engines, see Figure 5. Both EVMP, ADVI and NUTS successfully converged to almost coinciding estimates of the transition matrix (no notable difference when visualized). We also show free energy tracks for EVMP and ADVI in Figure 5. In this experiment, Turing’s ADVI outperformed ForneyLab’s EVMP in terms of total execution time and the free energy minimization. As a mitigating factor in this analysis, the pre-compilation of the message passing schedule in ForneyLab takes about 13 s, while actual execution of the generated inference algorithm is on par with Turing’s ADVI. Execution time details are shown in Table 2.

### 4.3. EVMP for a Switching State Space Model

In this experiment, we went beyond models that only contain continuously valued variables and inquired the capabilities of EVMP on a Switching State Space Model (SSSM) [27], which consists of both continuous and discrete hidden variables. The assumption of constant model parameters in the LDS of Section 4.2 does not account for the regime changes that occur in many dynamical systems of interest. The SSSM does allow for modeling parameter switches, and in this experiment we used the following model:

(24a) $$\begin{array}{cc} p(A) & =\prod_{k=1}^{3}\mathrm{Dir}\left(A\left[:,k\right];\alpha_{k}\right) \end{array}$$

(24b) $$\begin{array}{cc} p(z_{t}|z_{t-1}) & =\prod_{k=1}^{3}\prod_{j=1}^{3}A_{kj}^{z_{tk}z_{t-1,j}} \end{array}$$

(24c) $$\begin{array}{cc} p(x_{t}|x_{t-1},z_{t}) & =\prod_{k=1}^{3}N{\left(x_{t}|x_{t-1},v_{k}\right)}^{z_{tk}} \end{array}$$

(24d) $$\begin{array}{cc} p(y_{t}|x_{t}) & =N(y_{t}|x_{t},1) \end{array}$$

In this system, $y_{t}\in R$ are observations, $x_{t}\in R$ is a continuously valued hidden state and $z_{t}$ is a one-hot coded three-dimensional selection variable, i.e., $z_{tk}\in \left\{0,1\right\}$ and $\sum_{k=1}^{3}z_{tk}=1$. The parameters of the system are the state variances $v_{k}$ and concentration parameters $\alpha_{k}$. The elements of $\alpha_{k}$ are all 1, except the $k^{\mathrm{th}}$ element which is set to 100 to disfavor frequent regime switches, e.g., $\alpha_{2}={\left[1,100,1\right]}^{⊺}$.

**Figure 6:**
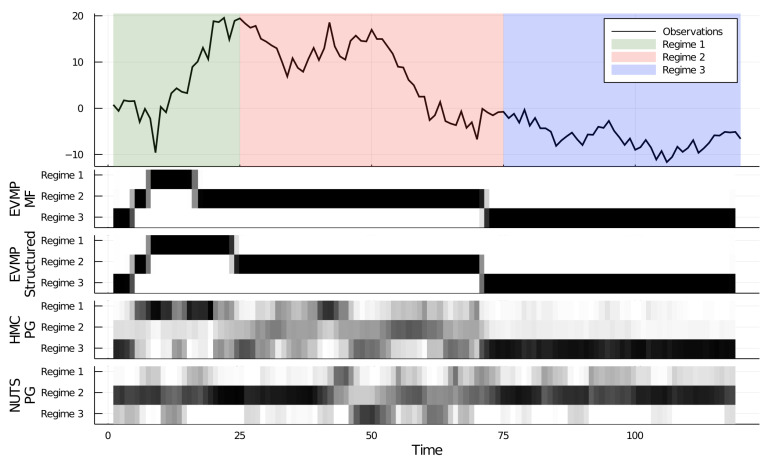
Performance results for automated inference in SSSM. **Top**: generated data set. **Bottom** 4 subgraphs: posterior for regime selection variable $z_{t}$ by MF-EVMP, SMF-EVMP, HMC and NUTS procedures respectively. In the Turing simulations (HMC and NUTS), the number of particles in the Particle Gibbs sampler was set to 50. In the NUTS sampler, the adaptation step size is 1000 and the target accept ratio is 0.65. The HMC sampler was tried with varying step sizes, including 0.001, 0.01, 0.1, 0.2, and 0.4 and leapfrog step numbers 10, 20, and 30. The best results are shown.

We generated T=120 data points from a random walk process (24c) and (24d) with process noise variance parameter $v=\left[v_{1},v_{2},v_{3}\right]=\left[10,4,1\right]$. From time step t=2 to t=25, we set $z_{t,1}=1$ and consequently $p\left(x_{t}|x_{t-1}\right)=N\left(x_{t-1},10\right)$. From time step t=26 to t=75, we set $z_{t,2}=1$ and between t=76 to t=T=120 we set $z_{t,3}=1$. The generated time series is shown in Figure 6.

The main difficulty in state inference for the SSSM stems from the coupling between *x* and *z*. This is because the variational message passing rules around the node $p(x_{t}|x_{t-1},z_{t})$ are not pre-defined in ForneyLab, although technically they can be worked out to closed-form expressions [27]. If EVMP were not available either, then a ForneyLab end user would be expected to manually derive closed-form update rules and implement these rules in an additional ForneyLab node. This type of manually assisted inference by end user calculations is what we try to avoid with EVMP and with probabilistic programming packages in general. EVMP enables the user to compensate for the lack of stored message-passing rules by introducing an auxiliary variable *s* in the model with a deterministic relation between *s* and *z*:

(25a) $$\begin{array}{cc} g(z_{t}) & =\sum_{k=1}^{3}z_{tk}\cdot v_{k} \end{array}$$

(25b) $$\begin{array}{cc} p(s_{t}|z_{t}) & =\delta (s_{t}-g\left(z_{t}\right)) \end{array}$$

(25c) $$\begin{array}{cc} p(x_{t}|x_{t-1},s_{t}) & =N(x_{t};x_{t-1},s_{t}) \end{array}$$

(25d) $$\begin{array}{cc} p(x_{t}|x_{t-1},z_{t}) & =\int p\left(x_{t}|x_{t-1},s_{t}\right)p\left(s_{t}|z_{t}\right)ds_{t}. \end{array}$$

After we extend model specification (24a–d) by (25a–d), then ForneyLab can run EVMP-based inference out of the box. Note that there is no need for manual inference calculations, but rather a simple manipulation of the generative model that makes the system suited for automated inference.

We tested the performance of two different constraints on the posterior distribution: (1) a mean-field assumption, i.e., $q\left(x_{1:T},z_{1:T}\right)=\prod_{t=1}^{T}q\left(x_{t}\right)q\left(z_{t}\right)$; (2) a *structured* mean-field assumption, i.e., $q\left(x_{1:T},z_{1:T}\right)=q\left(x_{1:T}\right)\prod_{t=1}^{T}q\left(z_{t}\right)$, see Figure 6. We observe that the structured factorization, being a less stringent constraint on *q*, yields a slightly better performance than the mean-field factorization, particularly in estimating the length of the first regime.

We also compared the performance of ForneyLab’s EVMP method to Turing’s inference method. As opposed to the previous two experiments, we could not use solely ADVI, nor Hamiltonian Monte Carlo (HMC, [28,29]) and NUTS samplers in this experiment since these procedures do not allow inference for discrete random variables. Turing does provide the option to use a Particle Gibbs (PG) sampler [30,31] for the estimation of the discrete random variables ($z_{1:T}$) in conjunction with the estimation of the continuous random variables ($x_{1:T}$, A) by HMC and NUTS. The performance results for NUTS-PG and HMC-PG are shown in Figure 6. The performance of the NUTS-PG and HMC-PG samplers in estimating the correct regimes is far below the EVMP results, although the HCM-PG sampler correctly identified the third regime. The run-time scores are shown in Table 3.

## 5. Related Work

Hybrid Monte Carlo variational inference techniques have been studied prior to our work. However, mainstream research predominantly consists of variational methods within Monte Carlo techniques as opposed to our Monte Carlo methods within a variational inference approach.

For instance, ref. [32] casts variational distributions as proposal distributions in a Markov-Chain Monte Carlo (MCMC) procedure. Similarly, ref. [33] employs variational methods to design adaptive proposal distributions for Importance Sampling (IS). In [34], gradient estimates of a variational objective are used to tune the parameters of the proposal distributions for MCMC. On the other hand, Monte-Carlo Co-Ordinate Ascent Variational Inference (MC-CAVI), proposed in [35], differs from the aforementioned methods in that it uses MCMC in the calculation of expectations required within the fixed-point iterations of Coordinate Ascent Variational Inference (CAVI).

In this paper, we follow a similar approach as [35], but we use IS to estimate the expectation quantities required in VMP. Both MCMC and IS have their own merits. IS smoothly interfaces with the message passing interpretation of Bayesian inference, which further leads to automated design of proposal distributions. We use Laplace approximation for Gaussian posteriors for variables with Gaussian priors. In the context of dynamical systems, this approach notably overlaps with Gaussian filtering techniques ([36], Section 6) that is often achieved by Assumed Density Filtering ([37], Section 8.4).

As we show in Appendix E, in the approach that we propose, it is also possible to run automated Bootstrap particle filtering [36,38] rather than Gaussian filtering methods. As show in [18], particle filtering can also be framed as message passing on a factor graph. The connection between the particle filtering and variational optimization was introduced in [39]. Their formalism is based on an extension of Particle Belief Propagation [40] to Tree-Reweighted Belief Propagation [41], while ours revolves around VMP. Similar to our approach, Particle Variational Inference (PVI) [42] aims at optimizing a variational objective by successive IS approximations to true posterior distributions. While PVI applies well to inference for discrete random variables, our EVMP proposal applies to both continuous and discrete random variables.

Variational inference in the context of deterministic building blocks in probabilistic models was studied in [43]. Wheras [43] allows non-linearities to be placed only after Gaussian nodes, the proposed EVMP method generalizes this concept to EF distributed factors.

Non-conjugate Variational Message Passing (NC-VMP) [44] addresses the non-conjugate factor issue in VMP. Assuming that the posterior distribution is an EF distribution, NC-VMP projects the messages to the distribution space of the posterior by equating their sufficient statistics. Thus NC-VMP tunes the natural parameters of the messages in such a way that they converge to the stationary points of the KL divergence between the approximated and true posteriors. Ref. [44] also reports that the algorithm necessitates damping for convergence in practice. In response, ref. [45] presents Conjugate-Computation Variational Inference (CVI) as a universal inference method that is based on stochastic optimization techniques. As opposed to alternative stochastic variational inference techniques, such as Black-Box Variational Inference [46] and Automatic Differentiation Variational Inference [21], CVI exploits the conjugacy structure of the probabilistic models, which leads to faster convergence. In CVI, non-conjugate factors are incorporated into coordinate ascent steps of mean-field variational inference (with ELBO objective) through a stochastic optimization procedure to form compact posterior approximations with standard probability distributions. In our EVMP approach, the Laplace approximation entails a similarly nested optimization procedure to form compact approximations with Gaussian distributions. Nevertheless, our particle approximations to the true posteriors obviate the need for additional gradient-based optimizations to estimate the parameters of the posteriors.

Finally, the original VMP paper [9] itself briefly mentions sampling methods to overcome the issues with non-conjugate priors. However, they do not extend this idea to deterministic nodes and rather present it as a fallback method whenever soft factors are tied to non-conjugate soft factor priors. Inspired by their vision of approximating the expectation quantities by sampling techniques, we introduce here a fully automated, very broadly applicable extended VMP procedure.

## 6. Discussion

In this paper, we present a method for almost universal variational inference on factorized probabilistic models. The core of our method is the locality feature of VMP: the messages at a soft factor are functions of expectations related to arguments of the factor. We employ IS to estimate these expectations or directly approximate posteriors by Laplace approximation if a Gaussian posterior is reasonable. We also extended the Julia package ForneyLab with the proposed EVMP method. In contrast to many alternative PPLs that are solely based on Monte Carlo methods, ForneyLab allows end users to take full advantage of closed-form message passing rules while resorting to small-scale numerical approximations only when needed. We showed that ForneyLab provides an efficient automated variational Bayesian inference tool that in some instances may be preferable to the state-of-the-art Turing package, especially for tasks that include filtering in dynamical models or discrete variables in state space models.

While the experiments support the notion that EVMP is a promising method for inference in non-linear and non-conjugate models, we have not tested our method yet in high-dimensional problems. It is well-known that importance sampling is not efficient in high dimensions [47]. Therefore, we anticipate that for high-dimensional inference tasks with continuous random variables, Hamiltonian Monte Carlo-based methods could outperform EVMP both in terms of run-time and quality of the estimates. Nevertheless, it should be possible to alleviate the deficiencies of EVMP in high dimensions by replacing IS and Laplace approximations by HMC samplers. In essence, HMC is an MCMC method and ref. [35] shows the efficiency of MCMC methods in estimation of the expectations that are required in variational inference. Yet, in lower dimensions, we favor IS and Laplace approximations both because of their promising performance scores in the experiments and also because EVMP relieves users of choosing hyperparameters for the best performance. Recall that in the SSSM experiments in Section 4.3, we tested HMC with various hyperparameters to attain the best performance, and yet EVMP was more successful in detecting the hidden regimes. Moreover, in contrast to EVMP, plain HMC is not applicable to estimate discrete variables and needs to be combined with other samplers to run inference on the models with discrete and continuous variables.

In Appendix C, we introduce a variational free energy estimation method that resorts to approximations only if the closed-form expressions of the information-theoretic measures are not available. This differs from alternative automated variational inference techniques, such as Automatic Differentiation Variational Inference (ADVI), which estimates the entire free energy over Monte Carlo summation. Moreover, like HMC, the applicability of ADVI is also limited to continuous variables.

In EVMP, proposal distributions for importance sampling are automatically set to forward messages. Although it is a practical solution with an elegant interpretation in a message passing context, forward messages do not carry information regarding observations. Therefore, we may not acquire useful samples from forward messages if the observations lead to peaky backward messages. In future work, we aim to investigate the effects of alternative proposal distribution design methods.

One major drawback of our ForneyLab implementation is that ForneyLab does not allow loops during the inference procedure. We rarely encounter this problem with soft factors since the mean field assumption breaks the loops by imposing additional factorizations in variational distributions. However, this may not be the case with deterministic nodes. This is because the input and the output variables of deterministic nodes are tied to each other through a deterministic mapping even after the mean field assumption. For example, consider the following mixture model specification: $p\left(z\right)=Ber\left(\rho \right),p\left(x|z\right)=\delta (x-g_{1}\left(z\right))$ with $g_{1}\left(z\right)=\mu_{1}\cdot z+\mu_{2}\cdot \left(1-z\right)$, $p\left(w|z\right)=\delta (w-g_{2}\left(z\right))$ with $g_{2}\left(z\right)=w_{1}\cdot z+w_{2}\cdot \left(1-z\right)$ and p(y|x,w)=N(x,1/w). Although it is a valid model specification with properly defined message passing rules, the EVMP algorithm is precluded due to the loop: the variable *z* is connected to two deterministic nodes (p(w|z) and p(x|z)) the outputs of which are connected to the same node p(y|x,w). Belief propagation (BP) [48,49] faces with a similar problem on loopy graphs. Nonetheless, it has been proven that iteratively running BP on loopy graphs often yields satisfactory approximations though the convergence is not guaranteed ([12], Section 8.4.7), ([37], Section 22.2). Therefore, it is worth investigating the performance of EVMP executed in a loopy setting.

There are similarities between EVMP and Expectation Propagation (EP) [50,51] in the sense that both methods estimate the moment parameters of posteriors. In contrast to EP, which approximates belief propagation (BP) [48,49] messages, EVMP approximates VMP messages, which is applicable to a broader range of model specifications. In future work, we aim to investigate and exploit this relation.

## 7. Conclusions

We developed a hybrid message passing-based approach to variational Bayesian inference that supports deterministic and non-conjugate model segments. The proposed Extended VMP (EVMP) method defaults to analytical updates for conjugate factor pairs and uses a local Laplace approximation or importance sampling when numerical methods are needed. EVMP was implemented in Julia’s ForneyLab package (see Appendix D) and a set of simulations shows very competitive inference performance on various inference tasks, particularly for state and parameter tracking in state-space models.

## Figures and Tables

**Table 1:** Run-time comparison of EVMP (in *ForneyLab.jl*) vs. ADVI (in *Turing.jl*) for the hierarchical Gaussian filter model.

Algorithm	Run Time (s)
EVMP (ForneyLab)	6.366±0.081
ADVI (Turing)	91.468±3.694

**Table 2:** Run-time results for transition matrix estimation in the LDS model.

Algorithm	Free Energy	Total Time (s)
EVMP (ForneyLab)	135.837	58.674±0.467
ADVI (Turing)	90.285	47.405±1.772
NUTS (Turing)	-	78.407±4.206

**Table 3:** Experimental results for switching state space model.

Algorithm	Free Energy	Total Time (s)
EVMP (Mean-field)	283.991	42.722±0.197
EVMP (Structured)	273.596	51.684±0.311
HMC-PG (Turing)	-	116.291±0.886
NUTS-PG (Turing)	-	51.715±0.441

**Figure A1:**
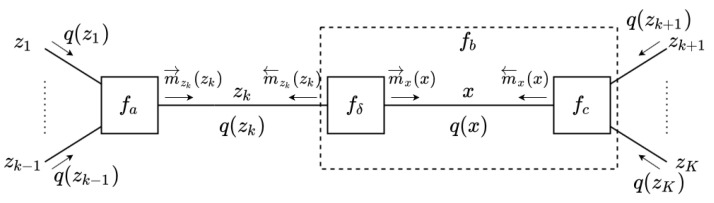
Deterministic conditional distributions often complicate VMP. An example deterministic conditional is visualized for $f_{\delta }\left(x,z_{k}\right)=p\left(x|z_{k}\right)=\delta \left(x-g\left(z_{k}\right)\right)$. $f_{\delta }\left(x,z_{k}\right)$ and $f_{c}\left(x,z_{k+1:K}\right)$ together form the composite node $f_{b}\left(z_{k:K}\right)$.

**Figure A2:**
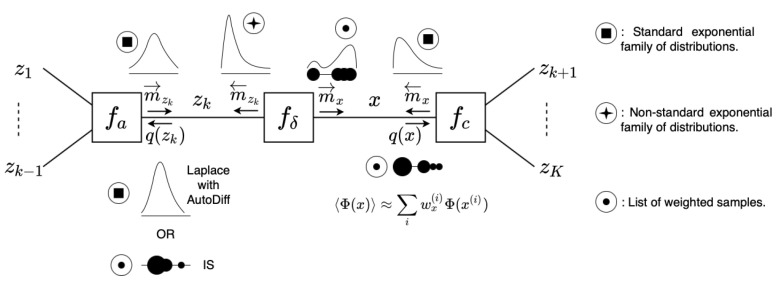
Messages around a deterministic node are visualized together with posterior approximations. In EVMP algorithm, forward messages from single input deterministic nodes are approximated by LWS representations. Backward messages, on the other hand, take non-standard exponential family distribution forms.

**Figure A3:**
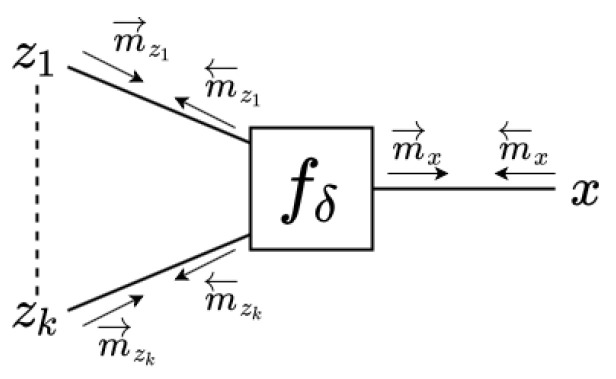
A deterministic node with inputs $z_{1},\ldots ,z_{k}$ and output *x*.

**Figure A4:**
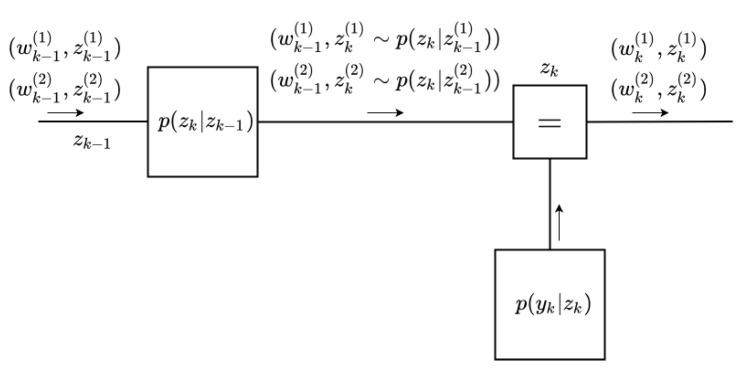
Bootstrap Particle Filtering employs the state transition distributions, $p(z_{k}|z_{k-1})$ as proposal distributions, which can easily be supported in our framework by defining BP rules at soft factors for incoming messages that are LWS. The rule is straightforward to implement: weights stay unchanged; for each incoming sample, instantiate a new conditional distribution and draw a sample from it. The weight update is automatically carried out at equality node by ${\tilde{w}}_{k}^{\left(i\right)}\propto p\left(y_{k}|z_{k}^{\left(i\right)}\right)w_{k-1}^{\left(i\right)}$, which is followed by a normalization: $w_{k}^{\left(i\right)}=\frac{{\tilde{w}}_{k}^{\left(i\right)}}{\sum_{j=1}{\tilde{w}}_{k}^{\left(j\right)}}$.

**Figure A5:**
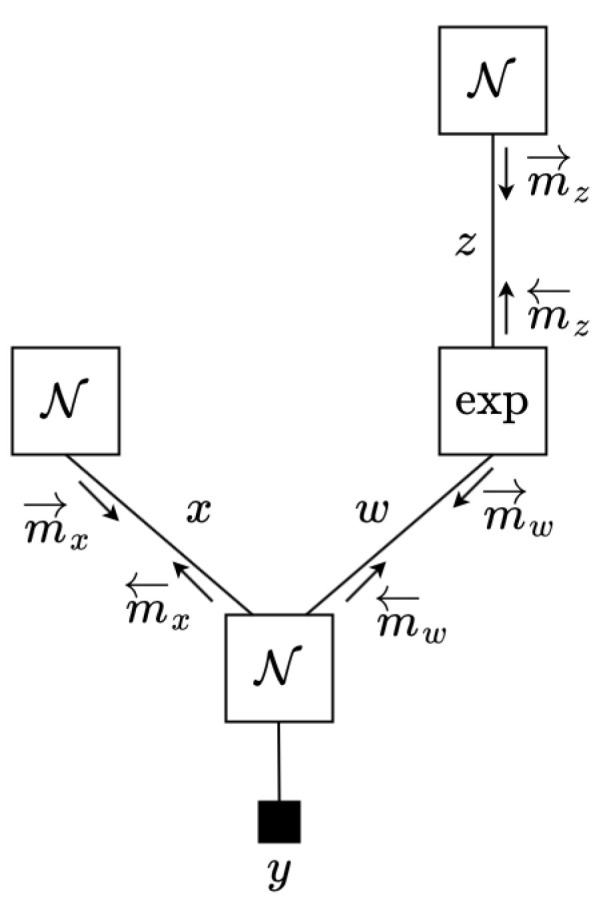
The model $p\left(x\right)=N\left(x;\mu_{x},v_{x}\right)$, $p\left(z\right)=N\left(z;\mu_{z},v_{z}\right)$, p(w|z)=δ(w−exp(z)), p(y|x,w)=N(y;x,1/w) is visualized together with the messages. The EVMP algorithm approximates the backward VMP message towards *x* by estimating $\langle w\rangle$ with Monte Carlo summation. Once this VMP message is approximated, the update for q(x) is available in closed form. The backward message toward *w* and *z* requires $\langle x\rangle$ and $\langle x^{2}\rangle$. These expectations can be computed analytically since they are the sufficient statistics of q(x). However, this time, the forward and the backward messages differ in sufficient statistics, which impedes the analytical calculations for q(w) and q(z). We approximate them by IS and Laplace, respectively.

## Data Availability

Not applicable.

## Abbreviations

The following abbreviations are used in this manuscript:

VMP	Variational Message Passing
EVMP	Extended Variational Message Passing
BP	Belief propagation
EP	Expectation propagation
FFG	Forney-style Factor Graph
EF	Exponential family
NEF	Non-standard exponential family
LWS	List of Weighted Samples
IS	Importance sampling
MCMC	Markov Chain Monte Carlo
HMC	Hamiltonian Monte Carlo
ADVI	Automatic Differentiation Variational Inference
PG	Particle Gibbs

## Appendix A. On the Applicability of VMP

In this section, we show that the applicability of the VMP algorithm relies on connected factors being conjugate pairs in exponential family of distributions. Non-conjugate connected factors lead to intractable posteriors and messages. Nevertheless, we show that for a given soft factor, the corresponding VMP messages are locally expressed in terms of some expectation quantities. If these expectations are not available in closed form, then we can estimate them to approximate the VMP messages around the non-conjugate factor pairs.

Let us focus on Figure 2. We postulate the following assumptions:

- $f_{a}\left(z_{1:k}\right)$ is an element of the exponential family (EF) of distributions, i.e.,
  (A1) $$\begin{array}{cc} f_{a}\left(z_{1:k}\right) & =p(z_{k}|z_{1:k-1})\\ \; & =h_{a}\left(z_{k}\right)\exp\left(\phi_{a}{\left(z_{k}\right)}^{⊺}\eta_{a}\left(z_{1:k-1}\right)-A_{a}\left(z_{1:k-1}\right)\right). \end{array}$$
  In this equation, $h_{a}\left(z_{k}\right)$ is a base measure, $\eta_{a}\left(z_{1:k-1}\right)$ is a vector of natural (or canonical) parameters, $\phi_{a}\left(z_{k}\right)$ are the sufficient statistics, and $A_{a}\left(z_{1:k-1}\right)$ is the log-partition function, i.e., $A_{a}\left(z_{1:k-1}\right)=\log\left(\int h_{a}\left(z_{k}\right)\exp\left(\phi_{a}{\left(z_{k}\right)}^{⊺}\eta_{a}\left(z_{1:k-1}\right)\right)dz_{k}\right)$. It is always possible to write the log-partition function as a function of natural parameters $A_{\eta_{a}}\left(\eta_{a}\right)$, such that $A_{\eta_{a}}\left(\eta_{a}\right)=A_{a}\left(z_{1:k-1}\right)$. Throughout the paper, we sometimes prefer the natural parameter parameterization of the log partition.
- We differentiate a few cases for $f_{b}$:
  1. $f_{b}$ is also an element of the EF, given by the following:
    (A2) $$\begin{array}{cc} f_{b}\left(z_{k:K}\right) & =p(z_{K}|z_{k:K-1})\\ \; & =h_{b}\left(z_{K}\right)\exp\left(\phi_{b}{\left(z_{K}\right)}^{⊺}\eta_{b}\left(z_{k:K-1}\right)-A_{b}\left(z_{k:K-1}\right)\right)\;, \end{array}$$
    and is a *conditionally conjugate pair* with $f_{a}$ for $z_{k}$. This (conditional conjugacy) property implies that, given $z_{k+1:K}$, we can modify $f_{b}$ in such a way that its sufficient statistics will match the sufficient statistics of $f_{a}$. Technically, this means we can rewrite $f_{b}$ as follows:
    (A3) $$\begin{array}{c} f_{b}\left(z_{k},z_{k+1:K}\right)=h_{b}\left(z_{K}\right)\exp\left(\phi_{a}{\left(z_{k}\right)}^{⊺}\eta_{ba}\left(z_{k+1:K}\right)+c_{ba}\left(z_{k+1:K}\right)\right). \end{array}$$
    The crucial element of this rewrite is that both $f_{a}\left(z_{1:k}\right)$ and $f_{b}\left(z_{k:K}\right)$ are written as exponential functions of the same sufficient statistics function $\phi_{a}\left(z_{k}\right)$. This case leads to the regular VMP update equations, see Appendix A.1.
    Our Extended VMP does not need this assumption and derives approximate VMP update rules for the following extensions.
  2. $f_{b}$ is an element of the EF, but not amenable to the modification given in (A3), i.e., it cannot be written as an exponential function of sufficient statistics $\phi_{a}\left(z_{k}\right)$. Therefore, $f_{b}$ is not a conjugate pair with $f_{a}$ for $z_{k}$.
  3. $f_{b}\left(z_{k:K}\right)$ is a composition of a deterministic node with an EF node, see Figure A1. In particular, in this case $f_{b}\left(z_{k:K}\right)$ can be decomposed as follows:
    (A4a) $$\begin{array}{cc} f_{b}\left(z_{k:K}\right) & =\int \delta \left(x-g\left(z_{k}\right)\right)f_{c}\left(x,z_{k+1:K}\right)dx \end{array}$$
    (A4b) $$\begin{array}{cc} \; & =f_{c}\left(g\left(z_{k}\right),z_{k+1:K}\right), \end{array}$$
    where $x=g(z_{k})$ is a deterministic, possibly nonlinear transformation and $f_{c}\left(x,z_{k+1:K}\right)$ is an element of the EF:
    (A5) $$\begin{array}{cc} f_{c}\left(x,z_{k+1:K}\right) & =p(z_{K}|x,z_{k+1:K-1})\\ \; & =h_{c}\left(z_{K}\right)\exp\left(\phi_{c}{\left(z_{K}\right)}^{⊺}\eta_{c}\left(x,z_{k+1:K-1}\right)-A_{c}\left(x,z_{k+1:K-1}\right)\right). \end{array}$$
    We assume that the conjugate prior to $f_{c}$ for random variable *x* has sufficient statistics vector ${\hat{\phi }}_{c}\left(x\right)$, and hence (A5) can be modified as follows:
    (A6) $$\begin{array}{c} f_{c}\left(x,z_{k+1:K}\right)=h_{c}\left(z_{K}\right)\exp\left({\hat{\phi }}_{c}{\left(x\right)}^{⊺}{\hat{\eta }}_{c}\left(z_{k+1:K}\right)+{\hat{c}}_{c}\left(z_{k+1:K}\right)\right), \end{array}$$
    where ${\hat{c}}_{c}\left(z_{k+1:K}\right)$ refers to the terms that does not include *x*.

### Appendix A.1. VMP with Conjugate Soft Factor Pairs

The original VMP algorithm arises as an efficient inference procedure in models that solely consist of conjugate factor pairs. This is because conjugate factor pairs yield analytically tractable messages and posterior calculations. Next, we shortly review the effect of conjugate factor pairs on VMP updates.

#### Appendix A.1.1. Messages and Posteriors

The VMP message from the factor $f_{a}$ to $z_{k}$ can easily be evaluated by applying (3) to (A1):

(A7) $$\begin{array}{cc} {\vec{m}}_{z_{k}}\left(z_{k}\right) & \propto h_{a}\left(z_{k}\right)\exp\left(\phi_{a}{\left(z_{k}\right)}^{⊺}{\langle \eta_{a}\left(z_{1:k-1}\right)\rangle }_{q(z_{1:k-1})}\right). \end{array}$$

Since $f_{b}$ is conjugate to $f_{a}$, its functional form can be modified as (A3) and by applying (3) to (A3), we find the VMP message from the factor $f_{b}$ to $z_{k}$:

(A8) $$\begin{array}{cc} {\overset{\leftarrow }{m}}_{z_{k}}\left(z_{k}\right) & \propto \exp\left(\phi_{a}{\left(z_{k}\right)}^{⊺}{\langle \eta_{ba}\left(z_{k+1:K}\right)\rangle }_{q(z_{k+1:K})}\right). \end{array}$$

Given that the messages ${\vec{m}}_{z_{k}}\left(z_{k}\right)$ and ${\overset{\leftarrow }{m}}_{z_{k}}\left(z_{k}\right)$ have the same sufficient statistics, the posterior update step reduces to summation of the messages’ natural parameters:

(A9) $$\begin{array}{c} q\left(z_{k}\right)\propto h_{a}\left(z_{k}\right)\exp\left(\phi_{a}{\left(z_{k}\right)}^{⊺}\underset{\eta_{ab}}{\underbrace{\left(\langle \eta_{a}\left(z_{1:k-1}\right)\rangle +\langle \eta_{ba}\left(z_{k+1:K}\right)\rangle \right)}}\right). \end{array}$$

For the sake of brevity, the distribution subscripts in the expectation notations are dropped. Note that we evaluate the posterior up to a normalization constant. Nevertheless, the log-normalizer function $A_{\eta_{a}}\left(\cdot \right)$ is readily available for EF distributions having sufficient statistics vector $\phi_{a}\left(z_{k}\right)$. As a consequence, the posterior evaluates to the following:

(A10) $$\begin{array}{c} q\left(z_{k}\right)=h_{a}\left(z_{k}\right)\exp\left(\phi_{a}{\left(z_{k}\right)}^{⊺}\eta_{ab}-A_{\eta_{a}}\left(\eta_{ab}\right)\right). \end{array}$$

Having showed that the conjugate factor pairs lead to a closed-form expression for posterior $q(z_{k})$, we now investigate which expectation quantities related to $q(z_{k})$ are required in the outgoing VMP messages from the factors $f_{a}$ and $f_{b}$ to, say $z_{1}$ and $z_{K}$:

(A11a) $$\begin{array}{cc} {\overset{\leftarrow }{m}}_{z_{1}}\left(z_{1}\right) & \propto \exp\left({\langle \log f_{a}\left(z_{1:k}\right)\rangle }_{q(z_{2:k})}\right), \end{array}$$

(A11b) $$\begin{array}{cc} {\vec{m}}_{z_{K}}\left(z_{K}\right) & \propto \exp\left({\langle \log f_{b}\left(z_{k:K}\right)\rangle }_{q(z_{k:K-1})}\right). \end{array}$$

In practice, the message ${\overset{\leftarrow }{m}}_{z_{1}}\left(z_{1}\right)$ is explicitly calculated by isolating the terms with $z_{1}$ in a sufficient statistics vector as it is done for $z_{k}$ in (A8). Similarly, ${\vec{m}}_{z_{K}}\left(z_{K}\right)$ is explicitly calculated, analogous to message ${\vec{m}}_{z_{k}}\left(z_{k}\right)$ in (A7). Here, we follow a rather different approach to explicitly show the expectations related to $q(z_{k})$ in the message calculations. Substituting $f_{a}$ and $f_{b}$ with (A1) and (A3) in (A11a,b), and keeping in mind that the mean-field assumption allows separation of the expectation quantities with distinct random variables, the messages evaluate to the following:

(A12a) $$\begin{array}{cc} {\overset{\leftarrow }{m}}_{z_{1}}\left(z_{1}\right) & \propto \exp\left({\langle \phi_{a}\left(z_{k}\right)\rangle }_{q(z_{k})}^{⊺}{\langle \eta_{a}\left(z_{1:k-1}\right)\rangle }_{q(z_{2:k-1})}-{\langle A_{a}\left(z_{1:k-1}\right)\rangle }_{q(z_{2:k-1})}\right), \end{array}$$

(A12b) $$\begin{array}{cc} {\vec{m}}_{z_{K}}\left(z_{K}\right) & \propto h_{b}\left(z_{K}\right)\exp\left({\langle \phi_{a}\left(z_{k}\right)\rangle }_{q(z_{k})}^{⊺}{\langle \eta_{ba}\left(z_{k+1:K}\right)\rangle }_{q(z_{k+1:K-1})}+{\langle c_{ba}\left(z_{k+1:K}\right)\rangle }_{q(z_{k+1:K-1})}\right). \end{array}$$

Notice that both messages require the expectation of the sufficient statistic vector $\phi_{a}\left(z_{k}\right)$. Fortunately, in EF distributions, ${\langle \phi_{a}\left(z_{k}\right)\rangle }_{q(z_{k})}$ is available in closed-form as the gradient of the log-normalizer ([52], Proposition 3.1):

(A13) $$\begin{array}{c} {\langle \phi_{a}\left(z_{k}\right)\rangle }_{q(z_{k})}=\nabla_{\eta_{a}}A_{\eta_{a}}{|}_{\eta_{a}=\eta_{ab}}. \end{array}$$

For the sake of completeness, we now show that this equality holds. Recall that $A_{\eta_{a}}\left(\eta_{a}\right)=\log\left(\int h_{a}\left(z_{k}\right)\exp\left(\phi_{a}{\left(z_{k}\right)}^{⊺}\eta_{a}\right)dz_{k}\right)$. Then,

(A14) $$\begin{array}{cc} \nabla_{\eta_{a}}A_{\eta_{a}}\left(\eta_{a}\right) & =\frac{\nabla_{\eta_{a}}\int h_{a}\left(z_{k}\right)\exp\left(\phi_{a}{\left(z_{k}\right)}^{⊺}\eta_{a}\right)dz_{k}}{\int h_{a}\left(z_{k}\right)\exp\left(\phi_{a}{\left(z_{k}\right)}^{⊺}\eta_{a}\right)dz_{k}}\\ \; & =\frac{\int \phi_{a}\left(z_{k}\right)h_{a}\left(z_{k}\right)\exp\left(\phi_{a}{\left(z_{k}\right)}^{⊺}\eta_{a}\right)dz_{k}}{\exp(A_{\eta_{a}}\left(\eta_{a}\right))}\\ \; & =\int \phi_{a}\left(z_{k}\right)h_{a}\left(z_{k}\right)\exp\left(\phi_{a}{\left(z_{k}\right)}^{⊺}\eta_{a}-A_{\eta_{a}}\left(\eta_{a}\right)\right)dz_{k}. \end{array}$$

Evaluating this gradient at $\eta_{a}=\eta_{ab}$, we reach the following:

(A15) $$\begin{array}{cc} \nabla_{\eta_{a}}A_{\eta_{a}}{|}_{\eta_{a}=\eta_{ab}} & =\int \phi_{a}\left(z_{k}\right)\underset{q(z_{k})}{\underbrace{h_{a}\left(z_{k}\right)\exp\left(\phi_{a}{\left(z_{k}\right)}^{⊺}\eta_{ab}-A_{\eta_{a}}\left(\eta_{ab}\right)\right)}}dz_{k}\\ \; & ={\langle \phi_{a}\left(z_{k}\right)\rangle }_{q(z_{k})}. \end{array}$$

#### Appendix A.1.2. Free Energy

As in the message and the posterior calculations, conjugacy eases the free energy calculation. We investigate it for (5), the free energy terms that include $z_{k}$. $F_{k}$ is decomposed as follows:

(A16) $$\begin{array}{c} F_{k}=\underset{\mathrm{Average}\;\mathrm{energy}\;\mathrm{terms}}{\underbrace{-E_{q(z_{1:k})}\left[\log f_{a}\left(z_{1:k}\right)\right]-E_{q(z_{k:K})}\left[\log f_{b}\left(z_{k:K}\right)\right]}}+\underset{\begin{array}{c} \mathrm{Negative}\;\mathrm{entropy} \end{array}}{\underbrace{E_{q(z_{k})}\left[\log q\left(z_{k}\right)\right].}} \end{array}$$

Substituting $f_{a}$, $f_{b}$ and $q(z_{k})$ with (A1), (A3) and (A10) in the above expression:

(A17) $$\begin{array}{cc} F_{k}= & -{\langle \log(h_{a}\left(z_{k}\right))\rangle }_{q(z_{k})}-{\langle \phi_{a}\left(z_{k}\right)\rangle }_{q(z_{k})}^{⊺}{\langle \eta_{a}\left(z_{1:k-1}\right)\rangle }_{q(z_{1:k-1})}+{\langle A_{a}\left(z_{1:k-1}\right)\rangle }_{q(z_{1:k-1})}\\ \; & -{\langle \log(h_{b}\left(z_{K}\right))\rangle }_{q(z_{K})}-{\langle \phi_{a}\left(z_{k}\right)\rangle }_{q(z_{k})}^{⊺}{\langle \eta_{ba}\left(z_{k+1:K}\right)\rangle }_{q(z_{k+1:K})}-{\langle c_{ba}\left(z_{k+1:K}\right)\rangle }_{q(z_{k+1:K})}\\ \; & +{\langle \log(h_{a}\left(z_{k}\right))\rangle }_{q(z_{k})}+{\langle \phi_{a}\left(z_{k}\right)\rangle }_{q(z_{k})}^{⊺}\eta_{ab}-A_{\eta_{a}}\left(\eta_{ab}\right). \end{array}$$

The expectation terms related to $z_{k}$ in $F_{k}$ are ${\langle \phi_{a}\left(z_{k}\right)\rangle }_{q(z_{k})}$ and ${\langle \log(h_{a}\left(z_{k}\right))\rangle }_{q(z_{k})}$. The former expectation is available in closed form, (A13). Thus $F_{k}$ is analytically tractable for those distributions that possess closed-form solution for ${\langle \log(h_{a}\left(z_{k}\right))\rangle }_{q(z_{k})}$.

In short, conjugate factor pairs facilitate the VMP procedure by allowing closed-form expressions for updates of messages (3), posteriors (4) and FE (5). Moreover, although exceptions exist, similar to the normalization of the posterior (A10), the messages ${\vec{m}}_{z_{k}}\left(z_{k}\right)$ and ${\overset{\leftarrow }{m}}_{z_{k}}\left(z_{k}\right)$ can be effortlessly normalized if the required expectations are known. Therefore, we can directly parameterize them with standard probability distributions and draw samples from them. This property of EF distributions plays a pivotal role in our automation of the importance sampling procedure.

### Appendix A.2. VMP with Non-Conjugate Soft Factor Pairs

Suppose that the soft factors $f_{a}$ and $f_{b}$ are no longer conjugate pairs, i.e., $f_{b}$ given in (A2) can be written in the following form:

(A18) $$\begin{array}{cc} f_{b}\left(z_{k:K}\right) & =h_{b}\left(z_{K}\right)\exp\left({\hat{\phi }}_{b}{\left(z_{k}\right)}^{⊺}{\hat{\eta }}_{b}\left(z_{k+1:K}\right)+{\hat{c}}_{b}\left(z_{k+1:K}\right)\right), \end{array}$$

where crucially ${\hat{\phi }}_{b}\left(z_{k}\right)\neq \phi_{a}\left(z_{k}\right)$. Notice that ${\hat{\eta }}_{b}\left(\neq \eta_{b}\right)$ is the natural parameters after the modification of (A2) to isolate the terms with $z_{k}$ in the sufficient statistics vector. Therefore, the messages ${\vec{m}}_{z_{k}}\left(z_{k}\right)$ and ${\overset{\leftarrow }{m}}_{z_{k}}\left(z_{k}\right)$ differ in sufficient statistics:

(A19a) $$\begin{array}{cc} {\vec{m}}_{z_{k}}\left(z_{k}\right) & \propto h_{a}\left(z_{k}\right)\exp\left(\phi_{a}{\left(z_{k}\right)}^{⊺}{\langle \eta_{a}\left(z_{1:k-1}\right)\rangle }_{q(z_{1:k-1})}\right), \end{array}$$

(A19b) $$\begin{array}{cc} {\overset{\leftarrow }{m}}_{z_{k}}\left(z_{k}\right) & \propto \exp\left({\hat{\phi }}_{b}{\left(z_{k}\right)}^{⊺}{\langle {\hat{\eta }}_{b}\left(z_{k+1:K}\right)\rangle }_{q(z_{k+1:K})}\right). \end{array}$$

In this case, the normalization constant calculation in the posterior update step (5) is not straightforward anymore; and worse, it is often intractable. The term *intractable* refers to integrals that are not available in closed-form for continuous variables. For discrete variables, it refers to summations that are not achievable in a feasible amount of time. The lack of the normalization constant, $\int {\vec{m}}_{z_{k}}\left(z_{k}\right){\overset{\leftarrow }{m}}_{z_{k}}\left(z_{k}\right)dz_{k}$, hinders the calculation of the expectations with $z_{k}$ terms that appear in out-going VMP messages from $f_{a}$ and $f_{b}$ to variables $z_{1:k-1}$ and $z_{k+1:K}$, respectively, e.g., ${\langle \phi_{a}\left(z_{k}\right)\rangle }_{q(z_{k})}$ and ${\langle {\hat{\phi }}_{b}\left(z_{k}\right)\rangle }_{q(z_{k})}$ in

(A20a) $$\begin{array}{cc} {\overset{\leftarrow }{m}}_{z_{1}}\left(z_{1}\right) & \propto \exp\left({\langle \phi_{a}\left(z_{k}\right)\rangle }_{q(z_{k})}^{⊺}{\langle \eta_{a}\left(z_{1:k-1}\right)\rangle }_{q(z_{2:k-1})}-{\langle A_{a}\left(z_{1:k-1}\right)\rangle }_{q(z_{2:k-1})}\right), \end{array}$$

(A20b) $$\begin{array}{cc} {\vec{m}}_{z_{K}}\left(z_{K}\right) & \propto h_{b}\left(z_{K}\right)\exp\left({\langle {\hat{\phi }}_{b}\left(z_{k}\right)\rangle }_{q(z_{k})}^{⊺}{\langle {\hat{\eta }}_{b}\left(z_{k+1:K}\right)\rangle }_{q(z_{k+1:K-1})}+{\langle {\hat{c}}_{b}\left(z_{k+1:K}\right)\rangle }_{q(z_{k+1:K-1})}\right). \end{array}$$

As a result, non-conjugacies obstruct the VMP procedure by hampering closed-form expectation calculations. Bear in mind that even though VMP procedure is obstructed due to intractable expectations, the messages are distinctly fixed for soft factors as functions of certain expectation quantities that are supposed to be calculated over their arguments. We use this property in our Extended VMP method.

### Appendix A.3. VMP with Composite Nodes

In this subsection, we shed light on the issues with composite nodes that are constructed by composition of EF distribution soft factors and deterministic conditionals, i.e., the following:

(A21) $$\begin{array}{cc} f_{b}\left(z_{k:K}\right) & =\int f_{\delta }\left(x,z_{k}\right)f_{c}\left(x,z_{k+1:K}\right)dx, \end{array}$$

where $f_{\delta }\left(x,z_{k}\right)=p\left(x|z_{k}\right)=\delta \left(x-g\left(z_{k}\right)\right)$ is a deterministic conditional distribution. Composite nodes enable us to build almost arbitrary factor nodes. For example, a mixture likelihood distribution $p\left(y|z\right)=N{\left(y;\mu_{1},\sigma^{2}\right)}^{z}N{\left(y;\mu_{2},\sigma^{2}\right)}^{\left(1-z\right)}$ with *z* a one-hot coded selection variable, can be constructed by composing an EF soft factor, a Gaussian, with a deterministic factor as ∫p(y|x)p(x|z)dx, where $p\left(y|x\right)=N\left(y;x,\sigma^{2}\right)$ and $p\left(x|z\right)=\delta (x-z\mu_{1}-\left(1-z\right)\mu_{2})$. However, composite nodes impose new challenges on inference procedures.

Now, let us try to calculate $q(z_{k})$. The forward VMP message ${\vec{m}}_{z_{k}}\left(z_{k}\right)$ is given in (A19a). Suppose that the conjugate prior to EF soft factor $f_{c}$ for *x* has the sufficient statistics vector ${\hat{\phi }}_{c}\left(x\right)$ (see (A6)). Then, the VMP message from $f_{b}$ to $z_{k}$ is as follows:

(A22) $$\begin{array}{cc} {\overset{\leftarrow }{m}}_{z_{k}}\left(z_{k}\right) & \propto \exp\left({\langle \log\left(\int \delta \left(x-g\left(z_{k}\right)\right)\exp\left({\hat{\phi }}_{c}{\left(x\right)}^{⊺}{\hat{\eta }}_{c}\left(z_{k+1:K}\right)\right)dx\right)\rangle }_{q(z_{k+1:K})}\right)\\ \; & \propto \underset{\mathrm{BP}}{\underbrace{\int \delta \left(x-g\left(z_{k}\right)\right)\underset{\mathrm{VMP}:{\overset{\leftarrow }{m}}_{x}\left(x\right)}{\underbrace{\exp\left({\hat{\phi }}_{c}{\left(x\right)}^{⊺}{\langle {\hat{\eta }}_{c}\left(z_{k+1:K}\right)\rangle }_{q(z_{k+1:K})}\right)}}dx}}\\ \; & =\underset{{\overset{\leftarrow }{m}}_{x}\left(g\left(z_{k}\right)\right)}{\underbrace{\exp\left({\hat{\phi }}_{c}{\left(g\left(z_{k}\right)\right)}^{⊺}{\langle {\hat{\eta }}_{c}\left(z_{k+1:K}\right)\rangle }_{q(z_{k+1:K})}\right)}}. \end{array}$$

Note that the above message reduces to VMP message from $f_{c}$ to *x* followed by Belief Propagation (BP) [48,49]. The resulting backward message ${\overset{\leftarrow }{m}}_{z_{k}}\left(z_{k}\right)$ has the sufficient statistics vector ${\hat{\phi }}_{c}\left(g\left(z_{k}\right)\right)$. If ${\hat{\phi }}_{c}\left(\cdot \right)=\phi_{a}\left(g^{-1}\left(\cdot \right)\right)$, this case reduces to ordinary VMP as discussed in Appendix A.1; otherwise this case is a special case of Appendix A.2 and $q(z_{k})$ is not available in closed-form. Hence, the outgoing messages from the factor nodes $f_{a}$ and $f_{b}$:

(A23a) $$\begin{array}{cc} {\overset{\leftarrow }{m}}_{z_{1}}\left(z_{1}\right) & \propto \exp\left({\langle \phi_{a}\left(z_{k}\right)\rangle }_{q(z_{k})}^{⊺}{\langle \eta_{a}\left(z_{1:k-1}\right)\rangle }_{q(z_{2:k-1})}-{\langle A_{a}\left(z_{1:k-1}\right)\rangle }_{q(z_{2:k-1})}\right), \end{array}$$

(A23b) $$\begin{array}{cc} {\vec{m}}_{z_{K}}\left(z_{K}\right) & \propto \exp(\langle \log(\int \delta (x-g\left(z_{k}\right))\\ \; & \;\;\;\;h_{c}\left(z_{K}\right)\exp\left({\hat{\phi }}_{c}{\left(x\right)}^{⊺}{\hat{\eta }}_{c}\left(z_{k+1:K}\right)+{\hat{c}}_{c}\left(z_{k+1:K}\right)\right)dx)\rangle_{q(z_{k:K-1})})\\ \; & =h_{c}\left(z_{K}\right)\exp\left({\langle {\hat{\phi }}_{c}\left(g\left(z_{k}\right)\right)\rangle }_{q(z_{k})}^{⊺}{\langle {\hat{\eta }}_{c}\left(z_{k+1:K}\right)\rangle }_{q(z_{k+1:K-1})}+{\langle {\hat{c}}_{c}\left(z_{k+1:K}\right)\rangle }_{q(z_{k+1:K-1})}\right)\\ \; & =h_{c}\left(z_{K}\right)\exp\left({\langle {\hat{\phi }}_{c}\left(x\right)\rangle }_{q(x)}^{⊺}{\langle {\hat{\eta }}_{c}\left(z_{k+1:K}\right)\rangle }_{q(z_{k+1:K-1})}+{\langle {\hat{c}}_{c}\left(z_{k+1:K}\right)\rangle }_{q(z_{k+1:K-1})}\right) \end{array}$$

are intractable. The last line in the above derivations follows from the transformation of variables [53], i.e., $q\left(z_{k}\right)=q\left(x\right)\left|\frac{dx}{dz_{k}}\right|$, and expose the automatable nature of Variational Message Passing: the VMP message ${\vec{m}}_{z_{K}}\left(z_{K}\right)$ requires expectation quantities that are related to arguments of the soft factor $z_{K}$ is tied to, which is in this case $f_{c}$. Therefore, once the VMP message passing rule is defined for the factor $f_{c}$ as a function of its arguments, we can instantiate the messages by providing the required expectation quantities. For example, the required expectation quantities related to argument *x* are contained in the sufficient statistics vector ${\hat{\phi }}_{c}\left(x\right)$.

## Appendix B. Derivation of Extended VMP

Here, we show the details of our solution approach that is based on importance sampling (IS) and Laplace approximation. First, we address the issues with deterministic mappings of random variables. The resulting technique emerges as a remedy for non-conjugate soft factor pairs problem as well.

### Appendix B.1. Deterministic Mappings with Single Inputs

We first address the issues with single-input deterministic mappings and generalize our solution to multiple inputs later on. Consider the sub-graph given in Figure A1, where the deterministic conditional $p(x|z_{k})$ is defined as $f_{\delta }\left(x,z_{k}\right)=\delta \left(x-g\left(z_{k}\right)\right)$. As derived in (A23a,b), we need the expectations ${\langle \phi_{a}\left(z_{k}\right)\rangle }_{q(z_{k})}$ and ${\langle {\hat{\phi }}_{c}\left(x\right)\rangle }_{q(x)}$ to calculate VMP messages towards edges $z_{1:k-1}$ and $z_{k+1:K}$, respectively. Suppose that Φ(·) is an element in the sufficient statistic vectors $\phi_{a}\left(z_{k}\right)$ and ${\hat{\phi }}_{c}\left(x\right)$. Then we need to be able to calculate ${\langle \Phi (x)\rangle }_{q(x)}$ and ${\langle \Phi (z_{k})\rangle }_{q(z_{k})}$. Let us start with evaluating ${\langle \Phi (x)\rangle }_{q(x)}$ first:

$$\begin{array}{c} {\langle \Phi (x)\rangle }_{q(x)}=\int q\left(x\right)\Phi \left(x\right)dx=\int q\left(z_{k}\right)\Phi \left(g\left(z_{k}\right)\right)dz_{k}. \end{array}$$

The second equality in the above expression is due to the transformation of variables, i.e., $q\left(x\right)=q\left(z_{k}\right)\left|\frac{dz_{k}}{dx}\right|$ [53].

Substituting $q(z_{k})$ with (4) in the above integral yields the following:

(A24) $$\begin{array}{c} {\langle \Phi (x)\rangle }_{q(x)}=\int \frac{{\vec{m}}_{z_{k}}\left(z_{k}\right){\overset{\leftarrow }{m}}_{z_{k}}\left(z_{k}\right)}{\int {\vec{m}}_{z_{k}}\left(z_{k}\right){\overset{\leftarrow }{m}}_{z_{k}}\left(z_{k}\right)dz_{k}}\Phi \left(g\left(z_{k}\right)\right)dz_{k}, \end{array}$$

where ${\overset{\leftarrow }{m}}_{z_{k}}\left(z_{k}\right)={\overset{\leftarrow }{m}}_{x}\left(g\left(z_{k}\right)\right)$, as given in (A22). Recall from Appendix A.3 that the normalizer, $\int {\vec{m}}_{z_{k}}\left(z_{k}\right){\overset{\leftarrow }{m}}_{z_{k}}\left(z_{k}\right)dz_{k}$, is often hard to calculate analytically.

We use importance sampling [11,36] to approximate the integral in (A24):

(A25) $$\begin{array}{cc} {\langle \Phi (x)\rangle }_{q_{x}} & =\int \frac{\frac{{\vec{m}}_{z_{k}}\left(z_{k}\right){\overset{\leftarrow }{m}}_{x}\left(g\left(z_{k}\right)\right)\pi \left(z_{k}\right)}{\pi (z_{k})}}{\int \frac{{\vec{m}}_{z_{k}}\left(z_{k}\right){\overset{\leftarrow }{m}}_{x}\left(g\left(z_{k}\right)\right)\pi \left(z_{k}\right)}{\pi (z_{k})}dz_{k}}\Phi \left(g\left(z_{k}\right)\right)dz_{k}\\ \; & \approx \sum_{i=1}^{N}\underset{w^{\left(i\right)}}{\underbrace{\left[\frac{\frac{{\vec{m}}_{z_{k}}\left(z_{k}^{\left(i\right)}\right){\overset{\leftarrow }{m}}_{x}\left(g\left(z_{k}^{\left(i\right)}\right)\right)}{\pi (z_{k}^{\left(i\right)})}}{\sum_{j=1}^{N}\frac{{\vec{m}}_{z_{k}}\left(z_{k}^{\left(j\right)}\right){\overset{\leftarrow }{m}}_{x}\left(g\left(z_{k}^{\left(j\right)}\right)\right)}{\pi (z_{k}^{\left(j\right)})}}\right]}}\Phi \left(g\left(z_{k}^{\left(i\right)}\right)\right), \end{array}$$

where $z_{k}^{\left(i\right)}$ for i=1,…,N are drawn from the proposal distribution $\pi (z_{k})$, i.e., $z_{k}^{\left(i\right)}∼\pi \left(z_{k}\right)$. $g(z_{k}^{\left(i\right)})$ for i=1,…,N are particles and their corresponding weights are denoted by $w^{\left(i\right)}$.

The design of a good proposal distribution has a critical role in IS. First, it is supposed to be an easy-to-sample distribution. Secondly, its support is required to be no smaller than the support of ${\vec{m}}_{z_{k}}\left(z_{k}\right){\overset{\leftarrow }{m}}_{x}\left(g\left(z_{k}\right)\right)$ [36]. Lastly, the proposal distribution is desired to be a good representation of $q_{k}\left(z_{k}\right)=\frac{{\vec{m}}_{z_{k}}\left(z_{k}\right){\overset{\leftarrow }{m}}_{z_{k}}\left(z_{k}\right)}{\int {\vec{m}}_{z_{k}}\left(z_{k}\right){\overset{\leftarrow }{m}}_{z_{k}}\left(z_{k}\right)dz_{k}}$ to attain a fast convergence [47]. In our automated design, ${\vec{m}}_{z_{k}}\left(z_{k}\right)$ constitutes the proposal distribution. Our choice is not optimal in a sense that information regarding the evidence is most often carried out by the backward message and it is not incorporated in our proposal design. However, ${\vec{m}}_{z_{k}}\left(z_{k}\right)$ satisfies the first two conditions since the messages are parameterized with standard distributions (easy-to-sample) and it has nonzero probability everywhere that the posterior has, too. Substituting $\pi (z_{k})$ with ${\vec{m}}_{z_{k}}\left(z_{k}\right)$ in (A25) yields the following:

(A26) $$\begin{array}{c} {\hat{\langle \Phi (x)\rangle }}_{q(x)}=\sum_{i=1}^{N}\underset{w^{\left(i\right)}}{\underbrace{\left[\frac{{\overset{\leftarrow }{m}}_{x}\left(g\left(z_{k}^{\left(i\right)}\right)\right)}{\sum_{j=1}^{N}{\overset{\leftarrow }{m}}_{x}\left(g\left(z_{k}^{\left(j\right)}\right)\right)}\right]}}\Phi \left(g\left(z_{k}^{\left(i\right)}\right)\right), \end{array}$$

where $z_{k}^{\left(i\right)}∼{\vec{m}}_{z_{k}}\left(z_{k}\right)$ for i=1,…,N and ${\hat{\langle \Phi (x)\rangle }}_{q(x)}$ denotes our estimator for ${\langle \Phi (x)\rangle }_{q_{x}}$.

Let us summarize the procedure in (A26) to define our first set of rules related to the deterministic nodes. (A26) consists of samples that are drawn from ${\vec{m}}_{k}\left(z_{k}\right)$ and transformed through deterministic mapping g(.). We cast this process as the forward message ${\vec{m}}_{x}\left(x\right)$ calculation. Once the samples are transformed, i.e., $g(z_{k}^{\left(i\right)})$, the weights $w^{\left(i\right)}$ are determined over ${\overset{\leftarrow }{m}}_{x}\left(.\right)$. We interpret this process as the collision of the forward ${\vec{m}}_{x}\left(x\right)$ and the backward messages ${\overset{\leftarrow }{m}}_{x}\left(.\right)$; hence, we relate it to the posterior calculation. Setting $\Phi (g\left(z_{k}^{\left(i\right)}\right))$ to $\delta (x-g\left(z_{k}^{\left(i\right)}\right))$, our interpretation of message collision becomes obvious since $\Phi \left(g\left(z_{k}^{\left(i\right)}\right)\right):=\delta \left(x-g\left(z_{k}^{\left(i\right)}\right)\right)$ results in a Monte Carlo estimate for q(x). As a result, we introduce our first set of rules related to deterministic nodes and the posterior approximation at the output edge of the deterministic node:

(A27a) $$\begin{array}{cc} \; & {\vec{m}}_{x}\left(x\right)\approx \left\{\left(\frac{1}{N},g\left(z_{k}^{\left(1\right)}\right)\right),\ldots ,\left(\frac{1}{N},g\left(z_{k}^{\left(N\right)}\right)\right)\right\}, \end{array}$$

(A27b) $$\begin{array}{cc} \; & q\left(x\right)\propto {\vec{m}}_{x}\left(x\right)\cdot {\overset{\leftarrow }{m}}_{x}\left(x\right)\approx \left\{\left(w_{x}^{\left(1\right)},x^{\left(1\right)}\right),\ldots ,\left(w_{x}^{\left(N\right)},x^{\left(N\right)}\right)\right\}, \end{array}$$

(A27c) $$\begin{array}{cc} \; & \mathrm{where}\;z_{k}^{\left(i\right)}∼{\vec{m}}_{k}\left(z_{k}\right),x^{\left(i\right)}=g\left(z_{k}^{\left(i\right)}\right),w_{x}^{\left(i\right)}=\frac{{\overset{\leftarrow }{m}}_{x}\left(x^{\left(i\right)}\right)}{\sum_{j=1}^{N}{\overset{\leftarrow }{m}}_{x}\left(x^{\left(i\right)}\right)}. \end{array}$$

Here, we introduce the term *list of weighted samples* (LWS) to refer to the distributions that are represented by a set of samples and corresponding weights. Above, ${\vec{m}}_{x}\left(x\right)$ and q(x) are represented by LWS distributions.

Now, we turn our attention to calculation of $q(z_{k})$ and the expectation quantity ${\langle \Phi (z_{k})\rangle }_{q(z_{k})}$. For this task we have two different strategies: if ${\vec{m}}_{z_{k}}\left(z_{k}\right)$ is a Gaussian message, we approximate $q(z_{k})$ by Laplace approximation which is also automatable thanks to automatic differentiation and otherwise we follow the IS procedure introduced above. Let us go over them starting from the latter.

#### Appendix B.1.1. Non-Gaussian Case

This time we are supposed to evaluate ${\langle \Phi (z_{k})\rangle }_{q(z_{k})}$ so that the VMP messages toward $z_{1},\ldots ,z_{k-1}$ can be computed. Notice that the procedure is exactly same with (A25), except that the expectation quantity of interest, ${\langle \Phi (z_{k})\rangle }_{q(z_{k})}$, does not involve the deterministic mapping g(.), this time. Therefore, by using ${\vec{m}}_{z_{k}}\left(z_{k}\right)$ as the proposal distribution, we can estimate ${\langle \Phi (z_{k})\rangle }_{q(z_{k})}$ as the following:

(A28) $$\begin{array}{c} {\hat{\langle \Phi (z_{k})\rangle }}_{q(z_{k})}=\sum_{i=1}^{N}\underset{w^{\left(i\right)}}{\underbrace{\left[\frac{{\overset{\leftarrow }{m}}_{x}\left(g\left(z_{k}^{\left(i\right)}\right)\right)}{\sum_{j=1}^{N}{\overset{\leftarrow }{m}}_{x}\left(g\left(z_{k}^{\left(j\right)}\right)\right)}\right]}}\Phi \left(z_{k}^{\left(i\right)}\right). \end{array}$$

This gives us the second set of rules related to deterministic mappings. An element of this new set of rules is that the backward message is directly passed in probability distribution function (pdf) form:

(A29) $$\begin{array}{c} {\overset{\leftarrow }{m}}_{z_{k}}\left(z_{k}\right)={\overset{\leftarrow }{m}}_{x}\left(g\left(z_{k}\right)\right). \end{array}$$

Recall from Appendix A.1 that the messages used to carry standard EF distributions. Now, we make an exception and introduce ${\overset{\leftarrow }{m}}_{z_{k}}\left(z_{k}\right)$, which is no longer associated with any of the standard EF distributions. Nonetheless, ${\overset{\leftarrow }{m}}_{z_{k}}\left(z_{k}\right)$ takes an exponential form since ${\overset{\leftarrow }{m}}_{x}\left(\cdot \right)$ is an EF distribution (see (A22)). Therefore, we call ${\overset{\leftarrow }{m}}_{z_{k}}\left(z_{k}\right)$ a *non-standard exponential family* (NEF) distribution. Having defined the backward message, let us evaluate the posterior $q(z_{k})$. Similar to q(x), substituting $\Phi (z_{k})$ with $\delta (z_{k}-z_{k}^{\left(i\right)})$ in (A28) gives us a Monte Carlo estimate of $q(z_{k})$:

(A30a) $$\begin{array}{cc} \; & q\left(z_{k}\right)\propto {\vec{m}}_{z_{k}}\left(z_{k}\right)\cdot {\overset{\leftarrow }{m}}_{z_{k}}\left(z_{k}\right)\approx \left\{\left(w_{z_{k}}^{\left(1\right)},z_{k}^{\left(1\right)}\right),\ldots ,\left(w_{z_{k}}^{\left(N\right)},z_{k}^{\left(N\right)}\right)\right\}, \end{array}$$

(A30b) $$\begin{array}{cc} \; & \mathrm{where}\;z_{k}^{\left(i\right)}∼{\vec{m}}_{z_{k}}\left(z_{k}\right),w_{z_{k}}^{\left(i\right)}=\frac{{\overset{\leftarrow }{m}}_{z_{k}}\left(z_{k}^{\left(i\right)}\right)}{\sum_{j=1}^{N}{\overset{\leftarrow }{m}}_{z_{k}}\left(z_{k}^{\left(j\right)}\right)}. \end{array}$$

#### Appendix B.1.2. Gaussian Case

In FFGs, the models are often constructed in such a way that the most prevailing message types will be Gaussians. This is because Gaussian messages facilitate inference by allowing many inference related operations to be executed in closed form, such as summation, conditioning, scaling and shifting by constants, etc. In order to retain the computational advantages of Gaussian distribution, we take it as an implicit hint that the posterior distribution is *Gaussian-like*, if ${\vec{m}}_{z_{k}}\left(z_{k}\right)$ is a Gaussian message. Then, we use Laplace approximation ([12], Section 4.4) to approximate $q(z_{k})$ with $N(z_{k};\mu_{z_{k}},V_{z_{k}})$:

(A31a) $$\begin{array}{cc} \mu_{z_{k}} & =\arg\max_{z_{k}}\left(\log{\vec{m}}_{z_{k}}\left(z_{k}\right)+\log{\overset{\leftarrow }{m}}_{z_{k}}\left(z_{k}\right)\right), \end{array}$$

(A31b) $$\begin{array}{cc} V_{z_{k}} & ={\left(-\nabla \nabla_{z_{k}}\left(\log{\vec{m}}_{z_{k}}\left(z_{k}\right)+\log{\overset{\leftarrow }{m}}_{z_{k}}\left(z_{k}\right)\right){|}_{z_{k}=\mu_{k}}\right)}^{-1}, \end{array}$$

where $\nabla_{z_{k}}f$ denotes the gradient of *f* with respect to $z_{k}$ and $\nabla \nabla_{z_{k}}{f|}_{z_{k}=\mu_{k}}$ refers to the Hessian of *f* with respect to $z_{k}$ evaluated at $\mu_{k}$. Note that the gradient and the Hessian respectively reduce to the first and the second derivatives if $z_{k}$ is scalar. Laplace approximation is a mode-seeking algorithm. We use automatic differentiation (autodiff) [13] to evaluate the gradient $\nabla_{z_{k}}\left(\log{\vec{m}}_{z_{k}}\left(z_{k}\right)+\log{\overset{\leftarrow }{m}}_{z_{k}}\left(z_{k}\right)\right)$ and employ it in a gradient-ascent algorithm to seek the mode (we supply the implementation details in Appendix D). Once the mode is reached, we evaluate the Hessian at the mode to fit the variance term for our Gaussian approximation.

The assumption we make here that ${\vec{m}}_{z_{k}}\left(z_{k}\right)$ implies a *Gaussian-like* $q(z_{k})$ paves the way of automating many well known inference procedures achieved through Laplace approximation, such as Bayesian logistic regression ([37], Section 8.4), Laplace-Gaussian filtering and smoothing in state space models [54], Poisson Linear Dynamical Systems [55], etc. However, our assumption would not be appropriate for all configurations. For example, Gaussian prior on rate parameter of a Poisson distribution would result in an ambiguous posterior since the domain of the rate is the positive real numbers while the Gaussian approximated posterior has a support on the entire real axis. A better model specification could be achieved by mapping a Gaussian distributed random variable to the rate parameter through an inverse-link function, exp in this example. Likewise, a multi-modal backward message ${\overset{\leftarrow }{m}}_{z_{k}}\left(z_{k}\right)$ with a support on real numbers often yields a multi-modal posterior which can be better captured with particle methods. (In Appendix E, we show that it is possible to run particle filtering through Gaussian factor nodes in our technique.)

In summary, our method resorts to Laplace approximation to approximate $q(z_{k})$ with a Gaussian distribution whenever ${\vec{m}}_{z_{k}}\left(z_{k}\right)$ is Gaussian. Therefore, the user of our method must keep in mind the consequences of prior choices and build her model accordingly.

The overall procedure for single input deterministic functions is depicted in Figure A2. In the next subsection, we extend this procedure to multiple input deterministic mappings.

### Appendix B.2. Deterministic Mappings with Multiple Inputs

Consider the deterministic node, $f_{\delta }\left(x,z_{1:k}\right)=\delta \left(x-g\left(z_{1:k}\right)\right)$, given in Figure A3 where the inputs to the deterministic function g(.) are $z_{1},\ldots ,z_{k}$ and the output is *x*.

Before starting the discussion on the backward messages, let us define the forward message ${\vec{m}}_{x}\left(x\right)$. Analogous to the single input case, we define ${\vec{m}}_{x}\left(x\right)$ with an LWS as the following:

(A32) $$\begin{array}{cc} \; & {\vec{m}}_{x}\left(x\right)\approx \left\{\left(\frac{1}{N},g\left(z_{1:k}^{\left(1\right)}\right)\right),\ldots ,\left(\frac{1}{N},g\left(z_{1:k}^{\left(N\right)}\right)\right)\right\},\\ \; & \mathrm{where}\;z_{1}^{\left(i\right)}∼{\vec{m}}_{z_{1}}\left(z_{k}\right),\ldots ,z_{k}^{\left(i\right)}∼{\vec{m}}_{z_{k}}\left(z_{k}\right). \end{array}$$

Once the message is calculated as a set of equally weighted samples, we scale the weights according to the importance score of their corresponding samples to represent q(x):

(A33a) $$\begin{array}{cc} \; & q\left(x\right)\propto {\vec{m}}_{x}\left(x\right)\cdot {\overset{\leftarrow }{m}}_{x}\left(x\right)\approx \left\{\left(w_{x}^{\left(1\right)},x^{\left(1\right)}\right),\ldots ,\left(w_{x}^{\left(N\right)},x^{\left(N\right)}\right)\right\}, \end{array}$$

(A33b) $$\begin{array}{cc} \; & \mathrm{where}\;x^{\left(i\right)}=g\left(z_{1:k}^{\left(i\right)}\right),w_{x}^{\left(i\right)}=\frac{{\overset{\leftarrow }{m}}_{x}\left(x^{\left(i\right)}\right)}{\sum_{j=1}^{N}{\overset{\leftarrow }{m}}_{x}\left(x^{\left(i\right)}\right)}. \end{array}$$

Now, let us define the backward messages propagated by the deterministic node. The exact backward message towards one of the input variables, say $z_{k}$, is the following:

(A34) $$\begin{array}{cc} {\overset{\leftarrow }{m}}_{z_{k}}\left(z_{k}\right) & =\int \delta \left(x-g\left(z_{1:k}\right)\right){\vec{m}}_{z_{1}}\left(z_{1}\right)\ldots {\vec{m}}_{z_{k-1}}\left(z_{k-1}\right){\overset{\leftarrow }{m}}_{x}\left(x\right)dz_{1}\ldots dz_{k-1}dx. \end{array}$$

Unfortunately, the above integral is often intractable. Even if all the variables are discrete and integral is replaced by summation, it becomes intractable in practice as the number of variables increases. Here, we address this issue with two different approximation strategies. As it is in the above subsection, type of the approximation depends on the incoming messages to the deterministic node from the input edges: if the messages ${\vec{m}}_{z_{1}}\left(z_{1}\right),\ldots ,{\vec{m}}_{z_{k}}\left(z_{k}\right)$ are all Gaussian, we approximate the joint posterior distribution of $z_{1},\ldots ,z_{k}$ by a Gaussian distribution. Then, we calculate the backward messages over the approximated joint posterior and incoming messages. Otherwise, we use Monte Carlo summation. Let us start with the latter case.

#### Appendix B.2.1. Monte Carlo Approximation to the Backward Message

Monte Carlo approximation to the the integral in (A34) is

(A35) $$\begin{array}{c} {\overset{\leftarrow }{m}}_{z_{k}}\left(z_{k}\right)\approx \frac{1}{N}\sum_{i=1}^{N}{\overset{\leftarrow }{m}}_{x}\left(g\left(z_{1}^{\left(i\right)},\ldots ,z_{k-1}^{\left(i\right)},z_{k}\right)\right), \end{array}$$

where $z_{j}^{\left(i\right)}∼{\vec{m}}_{z_{j}}\left(z_{j}\right)$ for j=1,…,k−1.

Once the message ${\overset{\leftarrow }{m}}_{z_{k}}\left(z_{k}\right)$ is approximately calculated and propagated as an NEF distribution, $q(z_{k})$ is also approximated either by IS or Laplace, depending on the message type ${\vec{m}}_{z_{k}}\left(z_{k}\right)$ as it is discussed in Appendix B.1.

#### Appendix B.2.2. Gaussian Approximation to the Backward Message

The above procedure yields two consecutive approximation processes in the calculation of $q(z_{k})$. Considering that we assumed ${\vec{m}}_{z_{k}}\left(z_{k}\right)$ implies that $q(z_{k})$ is *Gaussian-like*, we can avoid the approximation in (A35) if all the incoming messages ${\vec{m}}_{z_{1}}\left(z_{1}\right),\ldots ,{\vec{m}}_{z_{k}}\left(z_{k}\right)$ are Gaussian. We achieve this by approximating the joint posterior $q(z_{1:k})$ with Laplace, followed by a marginalization to evaluate $q(z_{k})$ and ${\overset{\leftarrow }{m}}_{z_{k}}\left(z_{k}\right)\propto q\left(z_{k}\right)/{\vec{m}}_{z_{k}}\left(z_{k}\right)$.

More precisely, consider the incoming messages ${\vec{m}}_{z_{j}}\left(z_{j}\right)=N\left(z_{j};\mu {{}^{\prime }}_{z_{j}},V{{}^{\prime }}_{z_{j}}\right)$ for j=1,…,k. Note that these messages carry posterior beliefs on $z_{1},\ldots ,z_{k}$, which can be represented with a joint belief ${\vec{m}}_{z_{1:k}}\left(z_{1:k}\right)$ constituted by concatenation of $z_{1},\ldots ,z_{k}$: $z_{1:k}=z_{1}⊕z_{2}⊕\ldots ⊕z_{k}={\left[\begin{array}{ccc} z_{1} & \ldots & z_{k} \end{array}\right]}^{⊺}$:

(A36) $$\begin{array}{cc} {\vec{m}}_{z_{1:k}}\left(z_{1:k}\right) & =N(\mu {{}^{\prime }}_{z_{1:k}},V{{}^{\prime }}_{z_{1:k}})\\ \; & =N\left(\left[\begin{array}{c} \mu {{}^{\prime }}_{z_{1}}\\ \vdots \\ \mu {{}^{\prime }}_{z_{k}} \end{array}\right],\left[\begin{array}{ccc} V{{}^{\prime }}_{z_{1}} & \ldots & 0\\ \vdots & \ddots & \vdots \\ 0 & \ldots & V{{}^{\prime }}_{z_{k}} \end{array}\right]\right). \end{array}$$

Now, we approximate $q(z_{1:k})$ by a Gaussian distribution $N(\mu_{z_{1:k}},V_{z_{1:k}})$ with a La- place approximation:

(A37a) $$\begin{array}{cc} \mu_{z_{1:k}} & =\underset{z_{1:k}}{\mathrm{argmax}}\log{\vec{m}}_{z_{1:k}}\left(z_{1:k}\right)+\log{\overset{\leftarrow }{m}}_{x}\left(g\left(z_{1},\ldots ,z_{k}\right)\right), \end{array}$$

(A37b) $$\begin{array}{cc} V_{z_{1:k}} & ={\left(-\nabla \nabla_{z_{1:k}}\left(\log{\vec{m}}_{z_{1:k}}\left(z_{1:k}\right)+\log{\overset{\leftarrow }{m}}_{x}\left(g\left(z_{1},\ldots ,z_{k}\right)\right)\right){|}_{z_{1:k}=\mu_{z_{1:k}}}\right)}^{-1}. \end{array}$$

By marginalizing out $z_{1},\ldots z_{k-1}$, we find $q(z_{k})$:

(A38) $$\begin{array}{c} q\left(z_{k}\right)=\int q\left(z_{1:k}\right)dz_{1}\ldots dz_{k-1}=N\left(\mu_{z_{k}},V_{z_{k}}\right). \end{array}$$

Recall that $q\left(z_{k}\right)\propto {\vec{m}}_{z_{k}}\left(z_{k}\right){\overset{\leftarrow }{m}}_{z_{k}}\left(z_{k}\right)$. This yields the following backward message

(A39) $$\begin{array}{cc} {\overset{\leftarrow }{m}}_{z_{k}}\left(z_{k}\right) & \propto q\left(z_{k}\right)/{\vec{m}}_{z_{k}}\left(z_{k}\right)=N\left(\mu_{z_{k}}^{{}^{\prime \prime }},V_{z_{k}}^{{}^{\prime \prime }}\right), \end{array}$$

where $V_{z_{k}}^{{}^{\prime \prime }-1}=V_{z_{k}}^{-1}-V_{z_{k}}^{{}^{\prime }-1}$ and $V_{z_{k}}^{{}^{\prime \prime }-1}\mu_{z_{k}}^{{}^{\prime \prime }}=V_{z_{k}}^{-1}\mu_{z_{k}}-V_{z_{k}}^{{}^{\prime }-1}\mu {{}^{\prime }}_{z_{k}}$. Note that we intentionally parameterize the Gaussian backward message ${\overset{\leftarrow }{m}}_{z_{k}}\left(z_{k}\right)$ with a precision-weighted mean $V_{z_{k}}^{{}^{\prime \prime }-1}\mu_{z_{k}}^{{}^{\prime \prime }}$ and precision $V_{z_{k}}^{{}^{\prime \prime }-1}$. The canonical parameterization (weighted-mean and precision) brings computational advantages, especially in state space models, by avoiding certain matrix inversions [15]. The approach that we introduced in this section resembles Expectation Propagation (EP) [50,51] in the sense that we first find the posterior, $q(z_{k})$, and then the backward message is evaluated by dividing the posterior to the incoming message. As it is stated in [51], Laplace Propagation [56] proposes an iterative Laplace approximation approach to mitigate intractable integral issues that sometimes emerge in EP.

So far, we have discussed how to extend VMP to those models with deterministic conditional distributions. To summarize, the resulting technique approximates the forward messages in deterministic nodes by LWS. Backward messages, on the other hand, are either directly propagated in NEF form or approximated with Gaussian distributions. We also showed posterior approximations related to these message types. In the next subsection, we attack the problem regarding the non-conjugate soft factor pairs.

### Appendix B.3. Non-Conjugate Soft Factor Pairs

Next, we address the problems defined in Appendix A.2. Consider the generic edge depicted in Figure 2. Suppose that the messages ${\vec{m}}_{z_{k}}\left(z_{k}\right)$ and ${\overset{\leftarrow }{m}}_{z_{k}}\left(z_{k}\right)$ differ in sufficient statistics and hence the normalization constant $\int {\vec{m}}_{z_{k}}\left(z_{k}\right){\overset{\leftarrow }{m}}_{z_{k}}\left(z_{k}\right)dz_{k}$ is analytically intractable. Recall that the very much same problem emerges in Appendix B.1 while calculating $q(z_{k})$. Therefore, the approximation rules defined in Appendix B.1 applies to non-conjugate factor pairs, as well. For the sake of comprehensiveness, the rules are summarized below.

1. If ${\vec{m}}_{z_{k}}\left(z_{k}\right)$ is a Gaussian message, apply Laplace to approximate $q(z_{k})$ with a Gaussian distribution as in (A31a,b).
2. Otherwise, use IS as in (A30a,b).

## Appendix C. Free Energy Approximation

Recall from Section 2 that variational inference transforms a difficult inference task to an easier optimization problem of a variational bound called the free energy F. Considering the fact that VMP converges to a stationary point by updating one posterior factor at a time, we anticipate that our approximations approach near the local optima.

As it is shown in Appendix A.1.2, the free energy is amenable to analytical calculations for those models that are solely comprised of conjugate factor pairs. The models that we address here do not allow the free energy to be calculated analytically. This is because analytically intractable expectation quantities, which complicates VMP in practice, also appear in the free energy calculation. Therefore, we provide an approximate free energy to the user so that they can track the convergence of the inference and also make a model comparison [9,57].

We introduce our free energy approximation approach over the sub-graph given in Figure A1, where $f_{a}\left(z_{1:k}\right)$ is a standard EF distribution (A1) and $f_{b}\left(z_{k:K}\right)$ is a composite node, i.e., $f_{b}\left(z_{k:K}\right)=\int \delta \left(x-g\left(z_{k}\right)\right)f_{c}\left(x,z_{k+1:K}\right)dx=f_{c}\left(g\left(z_{k}\right),z_{k+1:K}\right)$. Recall that $f_{c}\left(x,z_{k+1:K}\right)$ is modified as follows:

$$\begin{array}{c} f_{c}\left(x,z_{k+1:K}\right)=h_{c}\left(z_{K}\right)\exp\left({\hat{\phi }}_{c}{\left(x\right)}^{⊺}{\hat{\eta }}_{c}\left(z_{k+1:K}\right)+{\hat{c}}_{c}\left(z_{k+1:K}\right)\right). \end{array}$$

This sub-graph is a part of a larger FFG. First, we decompose the free energy as the following:

(A40) $$\begin{array}{c} F=\tilde{F}\underset{F_{k}}{\underbrace{\underset{\mathrm{Average}\;\mathrm{energy}\;\mathrm{terms}}{\underbrace{-E_{q(z_{1:k})}\left[\log f_{a}\left(z_{1:k}\right)\right]-E_{q(z_{k:K})}\left[\log f_{b}\left(z_{k:K}\right)\right]}}+\underset{\begin{array}{c} \mathrm{Negative}\\ \mathrm{entropy} \end{array}}{\underbrace{E_{q(z_{k})}\left[\log q\left(z_{k}\right)\right]}}}}, \end{array}$$

where $\tilde{F}$ stands for the free energy terms that are not subject to variables $z_{k}$. Explicitly writing the average energy terms, we have the following:

(A41a) $$\begin{array}{cc} -E_{q(z_{1:k})}\left[\log f_{a}\left(z_{1:k}\right)\right] & =-{\langle \log(h_{a}\left(z_{k}\right))\rangle }_{q(z_{k})}-{\langle \phi_{a}\left(z_{k}\right)\rangle }_{q(z_{k})}^{⊺}{\langle \eta_{a}\left(z_{1:k-1}\right)\rangle }_{q(z_{1:k-1})}\\ & \;+{\langle A_{a}\left(z_{1:k-1}\right)\rangle }_{q(z_{1:k-1})} \end{array}$$

(A41b) $$\begin{array}{cc} -E_{q(z_{k:K})}\left[\log f_{b}\left(z_{k:K}\right)\right] & =-\langle \log(\int \delta (x-g\left(z_{k}\right))\\ \; & \;\;\;h_{c}\left(z_{K}\right)\exp\left({\hat{\phi }}_{c}{\left(x\right)}^{⊺}{\hat{\eta }}_{c}\left(z_{k+1:K}\right)+{\hat{c}}_{c}\left(z_{k+1:K}\right)\right)dx)\rangle_{q(z_{k:K})}\\ \; & =-{\langle \log(h_{c}\left(z_{K}\right))\rangle }_{q(z_{K})}-{\langle {\hat{\phi }}_{c}\left(x\right)\rangle }_{q(x)}^{⊺}{\langle {\hat{\eta }}_{c}\left(z_{k+1:K}\right)\rangle }_{q(z_{k+1:K})}\\ \; & \;-{\langle {\hat{c}}_{c}\left(z_{k+1:K}\right)\rangle }_{q(z_{k+1:K})}. \end{array}$$

The above derivations closely follow the derivations in (A23a,b). Note that the expectation terms regarding $z_{k}$ in (A41b) are substituted by the expectations related to *x*, which are contained in the sufficient statistics vector ${\hat{\phi }}_{c}\left(x\right)$. This quantities are exactly same with the ones required to calculate VMP messages towards $z_{k+1:K}$, and we used IS to estimate them in (A26). Therefore, ${\langle {\hat{\phi }}_{c}\left(x\right)\rangle }_{q(x)}$ for the estimation of $-E_{q(z_{k:K})}\left[\log f_{b}\left(z_{k:K}\right)\right]$ is readily available.

Next, we investigate the terms related to $z_{k}$ in $-E_{q(z_{1:k})}\left[\log f_{a}\left(z_{1:k}\right)\right]$. Recall that for $q(z_{k})$, we have two approximation methods: (1) a Gaussian approximation to $q(z_{k})$ with Laplace, (2) an LWS approximation. $q(z_{k})$ is approximated with a Gaussian when ${\vec{m}}_{z_{k}}\left(z_{k}\right)$ is a Gaussian and this is the case if the factor node $f_{a}\left(z_{1:k}\right)=p\left(z_{k}|z_{1:k-1}\right)$ is a Gaussian distribution. In this case, $\phi_{a}\left(z_{k}\right)={\left[z_{k},z_{k}^{2}\right]}^{⊺}$ ($\phi_{a}\left(z_{k}\right)={\left[z_{k},z_{k}z_{k}^{⊺}\right]}^{⊺}$ for a multivariate Gaussian), $\log\left(h_{a}\left(z_{k}\right)\right)=-0.5\log\left(2\pi \right)$ ($\log\left(h_{a}\left(z_{k}\right)\right)=-0.5d\log\left(2\pi \right)$ for a d-dimensional multivariate Gaussian), and ${\langle \log(h_{a}\left(z_{k}\right))\rangle }_{q(z_{k})}$, ${\langle \phi_{a}\left(z_{k}\right)\rangle }_{q(z_{k})}$ are available in closed-form. Similarly, the entropy term $-E_{q(z_{k})}\left[\log q\left(z_{k}\right)\right]$ is available in closed-form for a Gaussian $q(z_{k})$. This completes the calculation of the expectation terms with $z_{k}$ in $F_{k}$.

In the case that $q(z_{k})$ is approximated with LWS as in (A30a,b), we approximate ${\langle \log(h_{a}\left(z_{k}\right))\rangle }_{q(z_{k})}$ and ${\langle \phi_{a}\left(z_{k}\right)\rangle }_{q(z_{k})}$ with IS as in (A28). Therefore, the approximations for the average energy terms are straightforward. For LWS approximated $q(z_{k})$, the main difficulty in the estimation of $F_{k}$ stems from the entropy calculation. This is because $\log(q\left(z_{k}\right))$ does not persist in functional form. The entropy approximation for weighted sample approximated distributions is often carried out by probability density estimates on weighted samples [58]. Fortunately, in our case, we do not need to fit a density estimate on LWS since the messages ${\vec{m}}_{z_{k}}\left(z_{k}\right)$ and ${\overset{\leftarrow }{m}}_{z_{k}}\left(z_{k}\right)$ afford the information regarding the density $q(z_{k})$. Let us derive an estimator for the entropy $H_{z_{k}}$:

(A42) $$\begin{array}{cc} H_{z_{k}} & =-\int q\left(z_{k}\right)\log q\left(z_{k}\right)dz_{k}\\ \; & =-\int q\left(z_{k}\right)\log\left(\frac{{\vec{m}}_{z_{k}}\left(z_{k}\right){\overset{\leftarrow }{m}}_{z_{k}}\left(z_{k}\right)}{\int {\vec{m}}_{z_{k}}\left(z_{k}\right){\overset{\leftarrow }{m}}_{z_{k}}\left(z_{k}\right)dz_{k}}\right)dz_{k}\\ \; & =\underset{H_{z_{k}}^{1}}{\underbrace{-\int q\left(z_{k}\right)\log\left({\vec{m}}_{z_{k}}\left(z_{k}\right){\overset{\leftarrow }{m}}_{z_{k}}\left(z_{k}\right)\right)dz_{k}}}\underset{H_{z_{k}}^{2}}{\underbrace{+\int q\left(z_{k}\right)\log\left(\int {\vec{m}}_{z_{k}}\left(z_{k}\right){\overset{\leftarrow }{m}}_{z_{k}}\left(z_{k}\right)dz_{k}\right)dz_{k}}}. \end{array}$$

We estimate the first term with the following Monte Carlo summation:

(A43) $$\begin{array}{c} {\hat{H}}_{z_{k}}^{1}=-\sum_{i=1}^{N}w_{z_{k}}^{\left(i\right)}\log\left({\vec{m}}_{z_{k}}\left(z_{k}^{\left(i\right)}\right){\overset{\leftarrow }{m}}_{z_{k}}\left(z_{k}^{\left(i\right)}\right)\right). \end{array}$$

The term with the log in $H_{z_{k}}^{2}$ is constant since $z_{k}$ is integrated out inside the log. Therefore $H_{z_{k}}^{2}$ simplifies further:

(A44) $$\begin{array}{cc} H_{z_{k}}^{2} & =\log\left(\int {\vec{m}}_{z_{k}}\left(z_{k}\right){\overset{\leftarrow }{m}}_{z_{k}}\left(z_{k}\right)dz_{k}\right)\underset{1}{\underbrace{\int q\left(z_{k}\right)dz_{k}}}. \end{array}$$

Recall from (A28) that the samples $z_{k}^{\left(1\right)},\ldots ,z_{k}^{\left(N\right)}$ are drawn from the message ${\vec{m}}_{z_{k}}\left(z_{k}\right)$. Therefore, the Monte Carlo estimate of $H_{z_{k}}^{2}$ is as follows:

(A45) $$\begin{array}{cc} {\hat{H}}_{z_{k}}^{2} & =\log\left(\frac{1}{N}\sum_{i=1}^{N}{\overset{\leftarrow }{m}}_{z_{k}}\left(z_{k}^{\left(i\right)}\right)\right). \end{array}$$

This completes the estimation of the terms with $z_{k}$ in $F_{k}$.

## Appendix D. Implementation Details in ForneyLab

Our extensions to VMP are readily available in ForneyLab [8], which is a Julia package for message passing based probabilistic programming. In this section, we provide the reader with some of the core implementation details and automation process of the method. First, the number of particles that commutes through deterministic nodes is set to 1000 by default. The user can change the number of samples during model specification. Similarly, the posterior of the variables that are connected to non-conjugate soft factor pairs are approximated by 1000 samples. For Laplace approximations, gradients are automatically calculated by automatic differentiation tools of Julia language. We use the *ForwardDiff* package [59] since it is a mature, universal automatic differentiation tool that aligns well with the needs of our approach.

## Appendix E. Bonus: Bootstrap Particle Filtering

Having implemented importance sampling to get around the complications in VMP, we now show how our technique inherently supports bootstrap particle filtering in state space models [36,38].

Recall that we automate Laplace approximation to retain the computational convenience of Gaussian filtering and smoothing. Although this choice sounds reasonable for those cases, we believe that the distributions over hidden states possess unimodal behavior, so it would not be sufficient to capture multi-modal distributions [36]. Similarly, due to non-linearities in the model specification and/or non-Gaussian process noise, Gaussian distribution might not be a plausible representation of the hidden states. In these cases, Sequential Monte Carlo methods [60] could be appealing because they flexibly recover asymmetric and mixture distributions.

In VMP setting, our method employs samples and their corresponding weights to deploy VMP messages which are parameterized by exponential family distributions. Alternatively, in Belief Propagation (BP) setting, a soft factor collects samples to instantiate the conditional distributions, and then draw samples from these conditionals. This process is depicted in Figure A4 with two samples for the sake of ease of visualization.

Having implemented the BP rule at a soft factor for incoming LWS messages, we have to show how posteriors are approximated through updating weights. Suppose that ${\vec{m}}_{z_{k}}\left(z_{k}\right)$ is a message that carries LWS, and ${\overset{\leftarrow }{m}}_{z_{k}}\left(z_{k}\right)$ is parameterized either by an EF or an NEF. Then, we define the posterior update rule as follows:

(A46a) $$\begin{array}{cc} \mathrm{Given}\;{\vec{m}}_{z_{k}}\left(z_{k}\right) & =\left\{\left(w_{k-1}^{\left(1\right)},z_{k}^{\left(1\right)}\right),\ldots ,\left(w_{k-1}^{\left(N\right)},z_{k}^{\left(N\right)})\right)\right\},\\ q(z_{k}) & \propto {\vec{m}}_{z_{k}}\left(z_{k}\right){\overset{\leftarrow }{m}}_{z_{k}}\left(z_{k}\right)\\ & \approx \left\{\left(w_{k}^{\left(1\right)},z_{k}^{\left(1\right)}\right),\ldots ,\left(w_{k}^{\left(N\right)},z_{k}^{\left(N\right)})\right)\right\}, \end{array}$$

(A46b) $$\begin{array}{cc} \mathrm{where}\; & w_{k}^{\left(i\right)}\propto w_{k-1}^{\left(i\right)}{\overset{\leftarrow }{m}}_{z_{k}}\left(z_{k}^{\left(i\right)}\right). \end{array}$$

In Bootstrap particle filtering, these rules update the weights at equality nodes, automatically. A major drawback of sequential importance sampling methods is that the further samples commute over time steps, the more they lose their ability to recover the underlying process, and many of the weights approach to zero. This phenomenon is known as the degeneracy problem and can be alleviated by resampling [36,60]. In our automated setting, at each weight update step, we measure the effectiveness of the existing samples by $n_{\mathrm{eff}}=\frac{1}{\sum_{i=1}^{N}{\left(w^{\left(i\right)}\right)}^{2}}$, as it is shown in [36]. Then, we resample if $n_{\mathrm{eff}}<N/10$ [36]. A user can effortlessly execute a particle filtering procedure in our method by putting an LWS prior on the first hidden state of a sequential model and running BP inference on the model (For demonstration purposes, we implemented BP rules at Gaussian node for LWS messages. The user can implement the very same rules for other soft factors according to their needs. Visit https://github.com/biaslab/ForneyLab.jl/blob/master/demo/bootstrap_particle_filter.ipynb (accessed on 25 June 2021) for a toy example.).

## Appendix F. Illustrative Example

Consider the following model visualized in Figure A5: $p\left(x\right)=N\left(x;\mu_{x},v_{x}\right)$, $p\left(z\right)=N\left(z;\mu_{z},v_{z}\right)$, p(w|z)=δ(w−exp(z)), p(y|x,w)=N(y;x,1/w), with observation *y*. In [9], VMP messages are provided as an example for a normal node parameterized by mean and precision. Here, we augment their example by a deterministic node to illustrate how Extended VMP operates to approximate the posteriors for *x*, *w* and *z*:

- Initiate q(x), q(z) by Normal distributions and q(w) by an LWS.
- Repeat until convergence the following three steps:
  1. - Choose *w* for updating.
    - Calculate VMP message ${\overset{\leftarrow }{m}}_{w}\left(w\right)$ by (14). In this case,
      $$\begin{array}{cc} {\overset{\leftarrow }{m}}_{w}\left(w\right) & \propto \exp\left({\left[\begin{array}{c} \log w\\ w \end{array}\right]}^{⊺}\left[\begin{array}{c} 0.5\\ \langle x\rangle y-0.5\left(\langle x^{2}\rangle +y^{2}\right) \end{array}\right]\right)\\ \; & \propto Ga\left(w;1.5,-\langle x\rangle y+0.5\left(\langle x^{2}\rangle +y^{2}\right)\right), \end{array}$$
      where Ga(x;α,β) is a Gamma distribution with shape α and rate β.
    - Calculate ${\vec{m}}_{w}\left(w\right)$ by the following (16):
      $$\begin{array}{cc} \; & {\vec{m}}_{w}\left(w\right)=\left\{\left(\frac{1}{N},\exp\left(z^{\left(1\right)}\right)\right),\ldots ,\left(\frac{1}{N},\exp\left(z^{\left(N\right)}\right)\right)\right\},\\ & \mathrm{where}\;z^{\left(i\right)}∼{\vec{m}}_{z}\left(z\right)=N\left(z;\mu_{z},v_{z}\right). \end{array}$$
    - Update q(w) by Section 3.5 rule (3).
  2. - Choose *z* for updating.
    - Calculate ${\overset{\leftarrow }{m}}_{z}\left(z\right)$ by (18), which is a NEF distribution:
      $$\begin{array}{c} {\overset{\leftarrow }{m}}_{z}\left(z\right)={\overset{\leftarrow }{m}}_{w}\left(\exp\left(z\right)\right)\propto \exp\left({\left[\begin{array}{c} z\\ \exp(z) \end{array}\right]}^{⊺}\left[\begin{array}{c} 0.5\\ \langle x\rangle y-0.5\left(\langle x^{2}\rangle +y^{2}\right) \end{array}\right]\right). \end{array}$$
    - The forward message is simply the prior: ${\vec{m}}_{z}\left(z\right)=N\left(z;\mu_{z},v_{z}\right)$
    - Update q(z) by Section 3.5 rule (2)(a).
  3. - Choose *x* for updating.
    - Calculate VMP message ${\overset{\leftarrow }{m}}_{x}\left(x\right)$ by (14). In this case,
      $$\begin{array}{cc} {\overset{\leftarrow }{m}}_{x}\left(x\right) & \propto \exp\left({\left[\begin{array}{c} x\\ x^{2} \end{array}\right]}^{⊺}\left[\begin{array}{c} \langle w\rangle y\\ -0.5\langle w\rangle \end{array}\right]\right)\\ \; & \propto N\left(x;y,1/\langle w\rangle \right). \end{array}$$
    - The forward message is the prior:
      $$\begin{array}{c} {\vec{m}}_{x}\left(x\right)=N\left(x;\mu_{x},v_{x}\right)\propto \exp\left({\left[\begin{array}{c} x\\ x^{2} \end{array}\right]}^{⊺}\left[\begin{array}{c} \mu_{x}/v_{x}\\ -0.5/v_{x} \end{array}\right]\right). \end{array}$$
    - Update q(x) by Section 3.5 rule (1), i.e., the following:
      $$\begin{array}{cc} q(x) & \propto \exp\left({\left[\begin{array}{c} x\\ x^{2} \end{array}\right]}^{⊺}\left[\begin{array}{c} \mu_{x}/v_{x}+\langle w\rangle y\\ -0.5(1/v_{x}+\langle w\rangle ) \end{array}\right]\right)\\ \; & =N\left(x;\frac{\mu_{x}+\langle w\rangle v_{x}y}{1+\langle w\rangle v_{x}},\frac{v_{x}}{1+\langle w\rangle v_{x}}\right). \end{array}$$

The expectation quantities $\langle w\rangle$, $\langle x\rangle$, $\langle x^{2}\rangle$ that appear in the message calculations are computed according to Section 3.8. Therefore, while $\langle w\rangle$ is estimated via a Monte Carlo summation, $\langle x\rangle$ and $\langle x^{2}\rangle$ are available in closed form.
